# Individual differences in navigation skill: towards reliable and valid measures

**DOI:** 10.1186/s41235-025-00642-5

**Published:** 2025-06-07

**Authors:** Jacob L. Lader, Kim V. Nguyen, Nora S. Newcombe

**Affiliations:** Department of Psychology and Neuroscience, Temple University, Philadelphia, PA USA

**Keywords:** Navigation, Spatial cognition, Virtual environment, Individual differences, Confirmatory factor analysis

## Abstract

Even though successful navigation is vital for survival, individuals vary widely in their navigation skills. Researchers have examined the correlates of such variation using a wide variety of paradigms. However, we know little about the relation among the paradigms used, and their validity for real-world behaviors. In this study, we assessed 94 young adult participants’ encoding of environmental features in one real-world and two virtual environments (or paradigms), using a within-subjects design. Each paradigm involved building a map from memory and pointing to the location of objects while standing at different locations in the environment. Two of the paradigms also used a route efficiency task in which participants aimed to take the shortest possible path to a target object. Factor analysis showed shared and unique variance in individual’s performance associated with each paradigm. Mental rotation and perspective taking tasks correlated with navigation performance differently for different paradigms. The data suggest that (1) virtual measures correlate with real-world ones, (2) the specific tasks used (pointing, map building, shortest route finding) are less important than the paradigm, and (3) there is common variance (i.e., shared individual differences) across paradigms. However, there is also unique paradigm-specific variation. Future research should use multiple paradigms to achieve reliable and valid assessments, ideally with shorter tasks for each.

**Supplementary Information:**

The online version contains supplementary material available at 10.1186/s41235-025-00642-5.

## Significance statement

Some people seem never to get lost, whereas others seem never to know exactly where they are, even in familiar territory. To understand the roots of these differences, and how to support poor navigators to develop better skills, we need to measure their ability. However, it is unclear whether we can assess navigation ability as a single dimension or whether there are several relevant dimensions, if skill(s) are uniform across environments, and whether virtual assessments relate to real-world behavior. Here, we evaluate these issues and provide evidence that, while navigation ability reflects important paradigm-specific contextual variance, there is also common variance at the paradigm level, including virtual and real-world. This finding suggests a common underlying cognitive component of spatial navigation that generalizes between paradigms but that is not fully explained only by the paradigm’s environments, modalities (e.g., virtual or real-world), or tasks. Future work should use more than one paradigm to ensure reliable and valid measures.

**Supplementary Information:**

The online version contains supplementary material available at 10.1186/s41235-025-00642-5.

## Introduction

Navigating from place to place is a basic cognitive function that we use in a variety of contexts, from walking home to piloting an aircraft. Individuals differ substantially in their navigational competencies (e.g., Ishikawa & Montello, 2006; Weisberg & Newcombe, 2018; Weisberg et al. 2014). A longstanding interest of navigation research is identifying the variables that might interact to produce individual patterns of navigation (e.g., Meneghetti et al., 2021; Wolbers & Hegarty, 2010). However, there are many different paradigms, all aiming at evaluating navigation behavior. Yet, there has been little research on whether there is consistency across paradigms, and whether inferences can be drawn from one study to another and to real-world behaviors (Newcombe et al., 2023). Lack of consensus greatly reduces the growth of knowledge. Shared measures would be useful for a range of aims from early detection of Alzheimer’s disease (Allison et al., 2016) to evaluation for pilot training.

Two well-explored distinct factors that may be required in real-world navigation are the skill to identify objects in rotated views (mental rotation; MRT) and to adopt different perspectives (perspective taking; PTT) (Hegarty & Waller, 2004; Wolbers & Hegarty, 2010). However, although mental rotation correlated with performance in route-learning (Galea & Kimura, 1993) and a variety of other navigation paradigms (e.g., Blajenkova et al., 2005; Moffat et al., 1998; Pazzaglia & DeBeni, 2006; Saucier et al., 2002), a review by Hegarty and Waller (2005) showed that MRT-navigation correlations are often not statistically significant. There is stronger support for the necessity of perspective taking in many navigational tasks (e.g., Allen et al., 1996; Fields & Shelton, 2006; Hegarty & Waller, 2004; Kozhevnikov et al., 2006; Muffato et al., 2020). Thus, though MRT and PTT are sometimes treated as a single latent factor (i.e., “visuospatial skills”) distinct from navigational measures (Hegarty et al., 2006; Meneghetti et al., 2021), MRT and PTT may variably affect navigation performance.

Another factor of interest in the field is whether people can accurately report their own navigational skills. Reliable and valid self-report measures of navigation could circumvent the resource demands of navigation testing (Weisberg et al., 2014). There are different kinds of self-report measures, including sense of direction (Hegarty et al., 2002, 2006; Kozlowski & Bryant, 1977; Sholl, 1988) and awareness of strategy and learning preferences (Brunec et al., 2018; Muffato et al., 2022; Pazzaglia & De Beni, 2001).

To study navigation behavior, subjects often learn an environment, with spaces ranging from open fields to enclosed mazes, and are tested on what they learned. This combination of environment, learning, and testing characteristics forms a paradigm. If navigation behavior measures a single underlying cognitive factor, there should be a significant degree of shared individual patterns of behavior across paradigms. Another possibility is that navigation behavior is explained by two factors rather than one, and that measures should be selected based on the nature of this dichotomy. One such two-factor explanation could have to do with the layout of the environment. For instance, large- versus small-scale or gridded versus non-gridded layout may differ from each other (Peer et al., 2021, 2024). Humans navigating in large open environments show behaviors consistent with using maps that include metric information, while humans navigating in confined and complex environments behave as if they are using non-Euclidean representations of space (Chrastil & Warren, 2014; Doner et al., 2023; Ericson & Warren, 2020; He & Brown, 2019; Moeser, 1988; Muryy & Glennerster, 2018; Zetzsche et al., 2009).

Another two-factor explanation is that there are separable factors for real-world versus desktop virtual environments (VE) or video-based navigation (Hegarty et al., 2006). This type of factor model would be based on sensory modality differences, since desktop VEs primarily provide visual information (i.e., visual modality) while more immersive environments also introduce vestibular and motor cues (i.e., vestibular/motor modality). Navigation may also refer to both locomotion and wayfinding factors (Montello, 2005), and virtual environments are not expected to capture both.

Despite these differences, the predominant argument for the use of VEs is that they are easy and cheap to run, easy to replicate, and likely give good approximations of real-world behavior. For example, Weisberg et al., (2014), Weisberg and Newcombe (2018) designed a paradigm to study route integration. It featured a virtual desktop computer replica of a real-world environment (RWE) used in a study conducted by Schinazi et al. (2013). Route integration itself was originally examined in the real world by Ishikawa and Montello (2006). A comparison of adult behavior between the virtual environment (VE) and RWE showed that learning is more accurate overall in the real world, but that the pattern of individual differences is similar (Weisberg et al., 2014). However, this comparison was between-subjects. Within-subject comparisons would allow us to determine whether individual performance carries over from one paradigm to another and is representative of performance in the real world (Hejtmanek et al., 2020). In a within-subject study, a smartphone/tablet wayfinding task accounted for similar, although smaller, adult (ages 18–35) variance as a like-designed wayfinding task in two different real-world cities (Coutrot et al., 2019). Using the same paradigm with older adult participants (ages 54–74), virtual navigation performance only predicted performance in the real-world for medium difficulty environments, not for environments that were easy or very difficult to navigate (Goodroe et al., 2024). Additionally, while the younger participants outperformed the older adults, they did not outperform them in the real world. In another study, despite indications that participants responded to the same features, albeit to different extents in a VE designed to closely replicate a RWE, performance on navigational tasks differed (Kalantari et al., 2024). This work seems to suggest that navigation is best explained by a two-factor model, but that there may be a high degree of shared variance in performance between RWEs and VEs.

Further complicating measure selection, it is also possible that different types of navigation tasks recruit distinct cognitive processes (Wiener et al., 2009), each of which could show individual differences (Weisberg et al., 2014). The traditional task classification is (a) knowledge about the destination and landmarks (b) knowledge about routes and (c) knowledge about spatial layout of areas (Golledge, 1999; Siegel & White, 1975; Van der Ham et al., 2020; Wiener et al., 2009). Developing on this multifactorial model, Van der Ham and Claessen (2020) showed that it is important to test several distinct functional domains of navigation ability; (a) knowledge of landmarks (b) knowledge of locations from the observers view (egocentric) (c) knowledge of paths between objects from a map perspective (allocentric) (d) knowledge of egocentric views along paths (e) allocentric knowledge of object locations. A key take away is that variance in behavior can be explained by selecting the right tasks. Depending on the degree of correlation between each factor, it is possible that individual tasks are measuring just one underlying cognitive factor from different directions. For instance, all tasks in a paradigm could correspond to use of a single cognitive representation.

Alternatively, rather than tasks, paradigms may best explain individual differences in navigation because of the myriads of choices that experimenters make to investigate a specific question about navigation. How do specific paradigms relate to each other and performance in the real-world? In other words, are they all representing the same ecologically valid navigation behavior or are their designs so specific that there is little correlation between them? For instance, some paradigms are about testing encoding of metric distances between objects in a rigid grid, such as along city blocks. Other paradigms are about testing awareness of relationships between objects in space without much rigid information in the environment. While they all measure navigation behavior, paradigm-specific variance may be inevitable.

Expanding on these findings, we addressed the question of real-world validity. Our objective was to establish the degree to which variance in navigation performance was shared between existing RWE and VE paradigms. In doing so, we also aimed to demonstrate the model that best explains this variance. The second aim was important because of the evidence in the existing literature that could account for unexplained variance between paradigms, aside from RWE versus VE. For any model that included more than one factor (e.g., one for RWE and one for VE), we had to establish the correlation between these factors. It was likewise important to establish whether the visuospatial cognitive skills and self-report measures were predictive of these model factors.

We drew several hypotheses from the existing literature about possible models that explain navigation behavior. One possibility was that a unifactorial model would explain the variance. We also investigated bifactorial models based on gridded versus non-gridded layouts or based on VE versus RWE. To explore the two-factor models, we selected two paradigms that used gridded environments (1 RWE and 1 VE), and one VE that used a non-gridded environment. Finally, we assessed two trifactorial models. In one model, across-paradigm factors were based on specific assessment tasks. We selected existing paradigms that included common tasks: pointing, map building, and route efficiency. Each of these tasks could be categorized according to function. The pointing task was used to test egocentric based knowledge about the relative locations of objects. The map-building task was used as a measure of the participants allocentric knowledge of the environment and its landmarks (Hegarty et al., 2006; Weisberg et al., 2014). The route efficiency task was used to test knowledge about paths between objects. The second trifactorial model examined if behavior aligns more with the paradigm in which participants learned and were tested on the environments as opposed to navigation being a function of the environmental features, tasks, or modality. The optimal trifactorial model would determine whether individual differences are best explained by the tasks used or by the specific paradigms.

We included commonly used versions of a MRT, a PTT, the Santa Barbara Sense of Direction (SBSOD) scale (Hegarty et al., 2002), and the Navigation Strategy Questionnaire (NSQ) (Brunec et al., 2018) to see whether these skills and self-report measures predict performance in the optimal model factors. If either visuospatial task predicts all the latent factors from the model, then this finding would further support the idea that it captures skills that are generally relevant to navigation. If either one predicts factors differently, then this finding might provide further insight into what aspects of navigational skill each captures. If either self-report measure predicts latent factors from the model, then it would support the validity of that self-report as an alternative measure of navigation ability.

We used a within-subjects design where participants encoded environmental features in the RWE and two VEs. We ran factor analysis with similar measures of behavior per paradigm with variables organized to test each of the possible patterns. Finally, we built linear models of factor scores to tie in paradigm-independent spatial measures (i.e., models visuospatial cognitive and self-report measures).

## Methods

### Participants

**Fig. 1:**
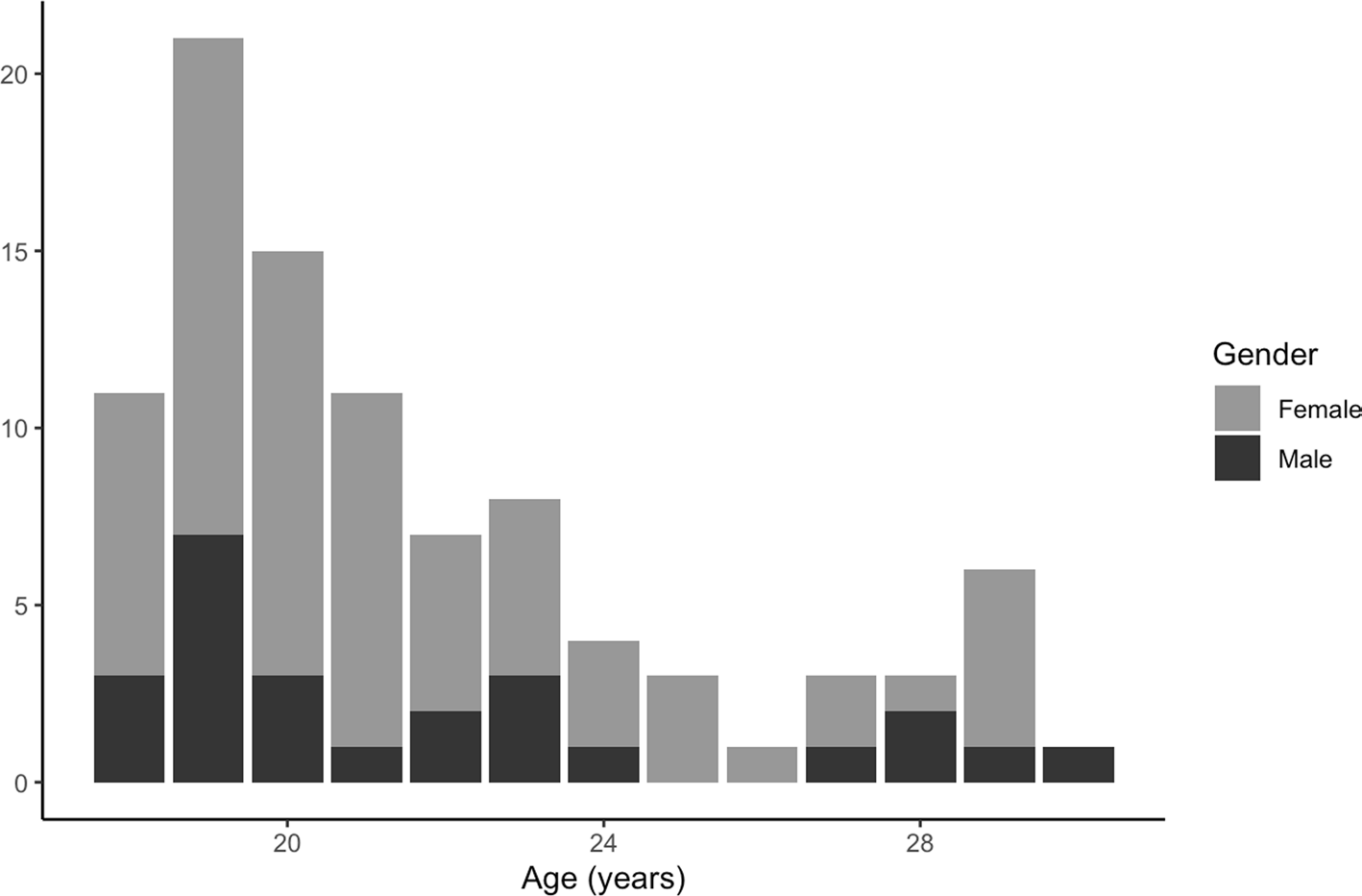
Age and reported gender breakdown

Ninety-four adults, aged 18–30 (M_age_ = 21.62 years, SD_age_ = 3.33, 69 females) (see Table 1 for demographic breakdown), participated in this study. We recruited participants in the city of Philadelphia by posting Facebook ads and physical flyers around campus. All participants completed online screening for their age, lack of psychological or developmental diagnoses, and fluency in English. If eligible, we scheduled participants for two 1.5-h sessions in the lab, on average 1.23 days apart and with no more than 9 days between the sessions. All participants consented and received monetary compensation (Fig. 1).

**Table 1:** Reported race, ethnicity, and gender demographics

Reported race/ethnicity	Reported gender		Total
	Female	Male	
Asian or Asian American	20	7	27
Black or African American	17	6	23
White	26	9	35
More than one race	3	1	4
Other or not reported	3	2	5
Hispanic ethnicity*	4	5	9
Total	69	25	94

*Subjects who reported ethnicity also reported race; therefore, ethnicity is not included in the total count

### Task design

### Santa Barbara sense of direction scale (SBSOD; Hegarty et al., 2002)

This was a self-report questionnaire designed to measure how strong of a navigator participants thought they were. Participants responded to statements on a seven-point Likert scale where 1 was “strongly agree” and 7 was “strongly disagree.” A response of 4 indicated that the participant neither agreed nor disagreed. The SBSOD had 15 questions and was untimed. Example statements were, “I have a poor memory for where I left things” and “I am very good at reading maps.” We calculated a score for each participant by first reverse scoring the positively phrased items. This way, all items were coded such that a high number indicated greater navigational accuracy and a low number indicated less accuracy. Next, we summed the scores for all items together, then divided the total by the number of items. The resulting overall average score of the participant, between 1 and 7, indicated perceived sense of direction, with 7 being strong.

### Mental rotation test (MRT; Vandenberg & Kuse, 1978)

We used a version of the MRT adapted by Peters et al. (1995). The MRT consisted of two parts and participants were given 3 min for 10 problems in each part with a break in between. The problems included a prompt image of several connected cubes. In each problem, participants had to choose two out of four objects that corresponded to the prompt image after being rigidly rotated (i.e., not undergoing any other geometric transformations). Participants received two points for each correct response but lost two points for any incorrect responses. No points were awarded or lost for missed items. Using this scoring method corrected for guessing.

### Paradigm 1: virtual SILC (spatial intelligence learning center) test of navigation (SILCton; Weisberg et al., 2014; Nazareth et al., 2018)

**Fig. 2:**
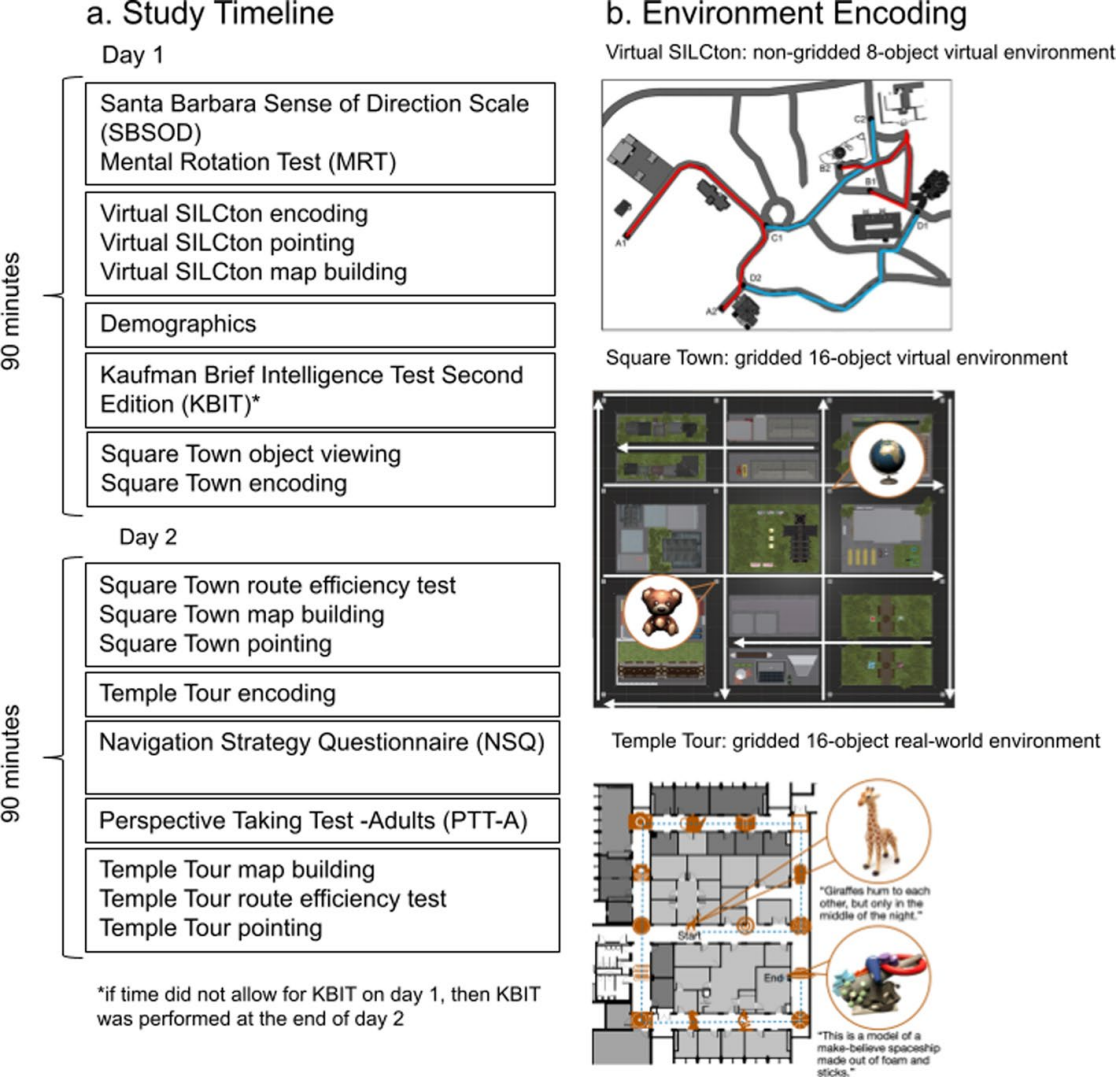
**a** Timeline of encoding and testing. First on day 1, was Virtual SILCton encoding and testing. Square Town encoding took place on day 1 and testing took place on day 2. All encoding and testing for Temple Tour were on day 2 after the Square Town testing. KBIT, demographics, SBSOD, MRT, NSQ, and PTT-A were distributed among day 1 and 2 as shown in the figure. Each session took up to 90 min. **b** Schematics of the environments for each of the three paradigms. Virtual SILCton was a virtual environment, where participants learned four paths and four building stimuli along the first two paths (in red as A and B, eight buildings total). The second two paths (in blue as C and D) were connecting routes. In Square Town, another virtual environment, participants learned eight paths between pairs of objects (16 object stimuli total). Square Town had a system of one-way roads (white arrows). Temple Tour was a real-world environment where participants learned 16 object stimuli along a tour path (blue dotted line)

Virtual SILCton encoding: In this part of the study, participants explored a virtual world (Fig. 2b) by walking along four different paths and learning the names and locations of buildings and how they relate to one another. Along each of the first two paths (in red; routes A and B), participants learned four buildings (eight buildings total). Each building was also indicated by a blue gem that hovered above the path. The gem helped ensure that participants did not pass by a building without making note of it. Next to each gem was a yellow and red sign with the name of the given building. The third and fourth routes (in blue; routes C and D) then provided opportunities to explore the environment from different perspectives, by connecting the first two routes to each other. Routes C and D did not contain any new buildings to learn, but some buildings from routes A and B were visible. Paths were indicated by large red arrows on the ground. All encoding trials were untimed, and participants moved through the environment at the same walking pace, traveling from start to finish and back to start on each route.

Before starting the virtual navigation, the experimenter also explained the controls to the participants and gave them the opportunity to practice the controls in the environment. Participants could control where they looked in the virtual environment by pointing with a computer mouse. While participants could look 360 degrees horizontally, they could not look more than 60 degrees vertically from their viewpoint parallel to the ground to simulate real-world head turning. Participants could also use keyboard keys to control their lateral movement. By using the mouse and keys in conjunction, participants could fully control their movement in the virtual world.

#### Virtual SILCton pointing

In this task, participants were placed at the start of route A, directly adjacent to the first building of that route. In the center of the screen was a crosshair. Participants could turn and point with the crosshair in any horizontal direction but were not able to move otherwise. At the top of the screen was a prompt with the name of one of the other seven buildings. Participants were told to rotate and point the crosshair in the direction of the named building and click once to record their answer. In some cases, the target building was visible from where the participant was standing, and in other cases it was not. The prompt at the top of the screen would change after each response, and participants then pointed to the new building.

Once participants pointed to all seven buildings from the first building, they were automatically dropped adjacent to another building on the same route and asked to point to the other seven buildings from there. They completed this task for all the buildings on route A and then participants were transported to route B and had to do the same pointing task for all the buildings there. The order of buildings that participants pointed to was random, but the locations from which they pointed were the same as the order by which participants learned the routes in. The pointing task was scored by measuring the smallest possible difference in degrees between the correct answer and each participant’s estimate, resulting in absolute angular error for each combination of buildings in the pointing task. We then averaged the angular error for judgments where the participant was pointing to a building on the same route as where they were pointing from to get a score of within-route average angular error. We also got a score of between-route average angular error by averaging the angular error for judgments where participants were prompted to point to a building on the route other than the one they were standing on. Guessing with no knowledge of the environment would yield an average score of 90°. We reverse scored the pointing errors by multiplying each score by − 1 (i.e., positive values are negative and vice versa), so that higher values indicated better performance, consistent with other tasks. Error could range from 0° to − 180° degrees.

#### Virtual SILCton map building

In the map-building task, participants were given a blank box on the computer screen representing a birds-eye view of the boundaries of the entire virtual environment. Below this image, participants saw aerial images of each of the eight buildings. The participants had to drag and drop each building to where they thought it was located in the virtual world by using the mouse. The map-building activity was untimed and there was no limit to how many times participants could move the buildings. The orientation of the buildings was fixed so that they could not be rotated, but participants were told that the buildings' orientation did not matter, only their relative location on the map. Accuracy on the map was measured using a bidimensional regression analysis to get the R^2^ (Carbon, 2013; Friedman & Kohler, 2003; Tobler, 1994). The R^2^ is the variance of the correct map that is explained by the participant’s map with scores closer to 1 indicating better mapping of object positions.

### Kaufman brief intelligence test 2nd edition (Kaufman & Kaufman, 2004)

This test was completed at the end of either the first or second session. Participants completed the verbal, riddles, and matrix reasoning subtests to generate a general IQ score (KBIT-IQ). Test stimuli were presented on a computer screen and riddles were read aloud by an experimenter. Participants started each test at their designated age start and continued until they got four consecutive trials wrong or until the end of the test.

### Paradigm 2: square town

#### Square town object viewing

Square Town used 16 computer generated three-dimensional models of familiar objects. Participants saw a digital slide show where they reviewed an image of each model and its name. Participants went through the slides at their own pace by pressing a key on the computer to go to the next image. Each object was viewed once.

#### Square town encoding

Square Town was a virtual city environment with four sides (Fig. 2b). The city had a different distinct landmark on each side: a desert, an iceberg, a meadow, and a canyon. It also had a system of one-way roads, indicated by arrows painted on the ground. Participants had to pay attention to this one-way system and were only allowed to travel in the indicated direction down each road. At each intersection in the city, there was an object, 16 objects total. These 16 objects were the same seen by participants in the object viewing task. The entire virtual city environment used a grid-like layout.

Participants were shown a short (~ 1 min) slide show with instructions for the encoding and route efficiency tasks. Participants learned eight routes, leading them between the different objects. Eight of the objects were start locations and eight of the objects were goal locations. In the encoding phase, participants saw green path markers guiding them along each route to the goal location object. The green path markers always showed the shortest possible route between each pair of objects while accounting for the one-way system. To travel, participants used the arrow keys for forward, backward, and turning movement with a fixed view (i.e., tank controls). They traveled along each of the eight paths once in the encoding phase and were untimed.

Routes were the same for all participants; however, the order the routes were shown in and the object locations on each route were controlled for order effects. Participants were randomly assigned to one of 49 conditions, with a max of two participants and a mean of 1.90 per condition. Each condition used a random route order and randomly placed different objects from the set of 16 in the same locations across conditions. Before participants started to travel on each route, they were prompted with the image and name of the goal object and told to look around to see what object they were standing next to at the start location. The name of the goal object was always visible at the top of the screen during the encoding and route efficiency trials.

#### Square town route efficiency

On day 2, participants did not have the green dots showing them where to go, and it was their job to find the shortest possible route to the goal object without breaking the one-way rule. The environment, routes, object locations, prompts, and controls were the same as on day 1, though the order the routes were shown in sometimes differed. There was no time limit for the route efficiency task. Each participant completed all eight routes and then repeated the task a second time. During the second route efficiency block, the order of the eight routes sometimes differed from the order in the first; however, everything else stayed the same. The participant location coordinates were recorded five times per second and used to calculate path distance for each trial of each block. We then summed the mean distance across all route efficiency trails per participant and divided that by a constant, the mean shortest distance, found by having an experimenter carefully follow the route encoding paths. The resulting score indicated how close participants were to the shortest possible distance (i.e., an error score), with 1 meaning they followed the shortest path exactly. It was possible for participants to score less than 1. This score would happen if participants traveled closer to corners along the learned routes, thereby shaving off distance during their route efficiency test. We reverse scored the efficiency error scores (i.e., positive values are negative and vice versa), so that higher values indicated better performance, consistent with other tasks.

#### Square town map building

In the map-building task, participants were given a blank box on the computer screen representing an aerial view of the boundaries of the entire virtual environment. The landmarks from the virtual environment were shown on each side of the box. Next to this image, participants saw images of each of the 16 objects they had seen in the Square Town environment. Participants had to drag and drop each object to where they thought it was located in the virtual world by using the mouse. The map-building activity was untimed and there was no limit to how many times participants could move the objects. Again, accuracy on the map was measured using a bidimensional regression analysis to get the R^2^ (Carbon, 2013; Friedman & Kohler, 2003; Tobler, 1994).

#### Square town pointing

In this task, participants completed 64 trials. Each trial, participants saw a certain view of the virtual world and were asked to imagine that they were standing next to the object they saw in that location. The participant indicated which direction they would go first to reach one of the other objects in the virtual world by clicking within one of the circles: forward, left/right, or backward. Each circle corresponded to a response in degrees, 0°, 90°, or 180°, respectively. Both left and right were scored as 90°. We calculated a mean error score for each participant by taking the absolute value of participant response minus the correct egocentric direction of the object in degrees (e.g., 0°, 90°, or 180°). We reverse scored the pointing errors (i.e., positive values are negative and vice versa), so that higher values indicated better performance, consistent with other tasks. Error could range from 0° to -180° degrees.

### Paradigm 3: temple tour

#### Stimuli norming

We used two sets of stimuli in this part of the experiment, each with 16 real-life objects. Sets A and B were actual objects participants saw in person during the encoding experiences. Each set had object categories that were semantically matched such that object A1 (basketball) semantically matched object B1 (volleyball). The similarity of pairs of objects was tested on an independent sample of 87 adults using Amazon Mechanical Turk on an IRB approved protocol. Participants completed the task online using Qualtrics and Pavlovia links and were compensated $5. Participants rated on a five-point Likert scale if the two images of the objects were dissimilar to similar based on how they perceived the two objects. Semantically matched pairs were consistently rated as highly similar while non-matched pairs were more dissimilar (e.g., volleyball and giraffe).

#### Temple tour encoding

Participants were led on an ~ 8-min tour by an experimenter during which they encoded 16 real-life objects and learned the spatial layout of the objects. This tour was set up on the 3rd floor of the psychology building at Temple University, Weiss Hall. The layout of the floor allowed for a grid patterned setup of the objects (Fig. 2b). Each object was set on a black stool in the same position for each participant. Object sets A and B were counterbalanced across participants, such that half the participants saw set A and half saw set B. Participants were instructed to remember what the objects looked like, the facts they heard, and where the objects were located while being led through a predetermined route. An experimenter accompanied the participant during the tour, keeping the pace at each object consistent by reading from a script. At each object, participants were told what the object was (e.g., stuffed giraffe), asked to examine and interact with the object, and then told a fun fact about the object (e.g., giraffes talk to each other by humming, but they only do it in the middle of the night). This task was designed to be a child-friendly version of the Baycrest Tour (Diamond et al., 2020).

#### Temple tour map building

Participants were told to recreate the tour environment from a bird’s-eye view by placing images of the 16 objects onto a blank white screen. All object images were visible all at once on the right side of the screen and could be moved freely. Participants pressed a key on the keyboard once they felt that their map was complete. The configuration of each participant’s map was compared to a correct map using bidimensional regression to get the R^2^ (Carbon, 2013; Friedman & Kohler, 2003; Tobler, 1994).

#### Temple tour route efficiency

Participants were led back into the environment where they started at one object and navigated to 12 other objects on the tour, sequentially (e.g., basketball to giraffe, giraffe to plant, plant to teapot, etc.). All objects remained in the same position as during encoding. They were asked to take the shortest route they could to each object. The researcher used pen and paper to record the routes traversed. Distance was measured by the total number of objects passed along a route over the least possible number of objects to pass (i.e., the shortest possible route). Efficiency scores closer to 1 were more efficient. In this paradigm, it was not possible to take a shortcut such that a score could be less than 1. We reverse scored the efficiency error scores (i.e., positive values are negative and vice versa), so that higher values indicated better performance, consistent with other tasks.

#### Temple tour pointing

Participants completed 10 trials of an onsite pointing task where they stood at one object and were told to face a cardinal direction. While facing this direction, participants were prompted to think about the location of a target object in the tour and then, indicate the direction of the target object using a 360º digital protractor (e.g., standing at the giraffe facing west, point to the record). Indicated angles and direction the protractor was turned (e.g., left or right) were recorded by the experimenter on paper. Angle was corrected for chirality, such that if the participant moved the protractor to the left, then their judgment was subtracted from 360°. Else, if they pointed the protractor to the right, then no change was made to their angular judgment. We made this correction because participants could have turned the protractor indirectly toward the target (e.g., turning the protractor left toward the target even though it would be more efficient to turn it rightward), resulting in an error despite pointing in the correct direction. For example, a leftward judgment of 315° is the same as a rightward judgment of 45º, though they would result in different error without the directional correction. Absolute angular error (|indicated angle–actual angle|) was then calculated for each trial. Angular difference was corrected to be below 180° by subtracting the result from 360 (e.g., |100°–295°|= 195°, then 360°–195° = 165). The resultant error scores were then averaged. Guessing with no knowledge of the environment would yield an average score of 90°. We reverse scored the pointing errors (i.e., positive values are negative and vice versa), so that higher values indicated better performance, consistent with other tasks. Error could range from 0° to − 180° degrees.

### Navigation strategy questionnaire (NSQ; Brunec et al., 2018)

This 14-item self-report survey was used to assess whether participants felt that they relied more on map-based or scene-based frames of reference when navigating. Participants responded to questions by selecting the radio button that best described how they feel they navigate. If none of the radio options applied, participants were asked to describe an answer in a space provided. To score the NSQ, we first converted the radio responses to numeric format to make subsequent categorization easier. Using an answer key, we then categorized responses depending on whether the response corresponded to map-based or scene-based navigation strategies. Alternative responses were not coded. The final score is the difference between the sums of each of the two categories (map–scene). A large positive score relays that a participant feels as though they rely more on map-based strategies, whereas a large negative score indicates greater reliance on scene-based strategies. A score of 0 indicates an equally perceived reliance on both strategies.

### Perspective taking test—adults (PTT-A; Brucato et al., 2023)

This computer task was adapted from a version for children (PTT-C; Frick et al., 2014), by presenting more difficult response options. In the PTT-A, participants were shown images of three-dimensional figurines holding cameras. In each image, the figurine was said to be taking a picture of three three-dimensional colored blocks. This task was presented in the form of a story where the figurines went to a museum of modern art and took pictures of the things they saw inside the museum. The participant had to choose how the picture the figurine was taking would look from where it was standing. Participants chose their response from among eight different object-arrangements that were simultaneously provided below the prompt image. There was no direct instruction for strategy use. Before starting the test, participants were led through an example problem by the experimenter, and then were able to complete a second practice problem on their own. The test itself contained 28 items presented in a fixed quasi-random order. Participants were allotted 3 min to complete all test items. Test items varied in the vantage point of the figurine around the display in 45° increments, with every angle presented four times. The objects' shape and color, the layout's orientation, and the figurine's gender were also counterbalanced among items. For each question a participant answered correctly, they would get a point. We scored this task by dividing the sum of points by the difference in minutes between the start and end time of the PTT-A recorded in seconds, since the total time would sometimes differ by a few seconds.

### Procedure

Figure 2a shows the timeline of the sessions relevant to this paper. We used a fixed order because there may be variance associated with balancing the order of paradigms (e.g., participant fatigue) that we are not interested in for this study. Day 1 took place entirely in the laboratory testing room. During day 1, participants first completed the SBSOD and the MRT. Next participants completed Virtual SILCton encoding, followed by the Virtual SILCton navigation tests (i.e., pointing and map building). After, they completed a short demographics questionnaire. If time allowed, participants also completed the KBIT-2, otherwise this task was administered on day 2. Finally, participants completed the Square Town object viewing and tour encoding.

The day 2 session started in the laboratory testing room with the Square Town tests (route efficiency test, map building, and pointing). This design was to maintain the original paradigm design, representative of experimental designs where encoding and testing happen on separate days. After completing the Square Town tests, participants were led into the hall where they completed Temple Tour encoding. This task was followed by the NSQ and the PTT-A in a laboratory testing room located at the end of the tour (to avoid exposing participants to object locations again). While in the testing room, they also completed the map-building activities for the Temple Tour. Then, participants went back into the Temple Tour environment for the route efficiency test and then pointing.

### Materials

Computer tasks on the Virtual SILCton website and the Qualtrics websites were completed in the Firefox browser on a 2020 MacBook Air (M1) with a 13.3-inch built-in Retina display running MacOS Ventura. The SBSOD and the MRT were run on the computer through the Virtual SILCton website, followed by Virtual SILCton itself. The same MacBook was used for the KBIT, NSQ, PTT-A, and Temple Tour map building. The NSQ and demographics were run on Qualtrics. The PTT-A was run on PsychoPy (Peirce et al., 2019).

All Square Town activities were done on an HP EliteBook 840 G5 running Windows 10 with an Intel Core i7-855OU processor, Intel UHD Graphics 620, and a 14-inch diagonal FHD IPS anti-glare LED-backlit display. Square Town encoding and route efficiency test were run on Unity version 2.4.2. The Square Town object viewing, map building, pointing, and the Temple Tour map building were also administered using PsychoPy.

Temple Tour encoding, route efficiency test, and pointing were all completed on the third floor of the Temple psychology building, Weiss Hall.

## Data analyses

All statistical analyses were completed in RStudio version 4.2.0 (R Core Team, 2024). All continuous variables were z-standardized. Gender was set as a categorical factor of 0 and 1, with 0 being male and 1 being female. The first step of analysis was to look at descriptive statistics to assess individual differences on the navigation paradigms.

Next, we did partial correlations to evaluate the internal consistency of each paradigm. We controlled for KBIT-IQ and age because they have been shown to influence measures of spatial knowledge, such as navigation performance. For instance, individual differences in spatial reasoning on environmental scales sometimes relate to nonspatial/verbal reasoning Cortes et al. (2022). There are also age-related differences in spatial navigation between younger and older adults (Moffat, 2009). Within-paradigm consistency tells us whether individuals perform in the same way across tasks. It does not tell us whether one task is a better measure than another. Finding internal consistency would suggest that tasks were measuring the same individual differences.

After within-paradigm correlations, we looked at cross-paradigm partial correlations. Cross-paradigm correlations could indicate consistency between the paradigms we used. Since we were interested in consistency at the paradigm level, we used composite scores. To do cross-paradigm partial correlations, we calculated three composite z-scores for each participant by averaging the three z-standardized measures within each paradigm. Z-standardization ensured that distribution differences between tasks would not mislead whether individuals performed in the same way between tasks. Correlations between these composite scores would convey that paradigms measure the same patterns in individual performance. We partialled out age and KBIT-IQ. Correlation between the VE and RWE would suggest that there is validity to the VEs used. This result would not tell us what leads this correlation.

Based on our five hypothesized models, we ran confirmatory factor analysis (CFA) to examine the statistical relationship between latent factors and their contributing variables. We conducted CFAs using the “lavaan” package in R (Rosseel, 2012). Prior to CFA, we isolated the nine variables (i.e., three measures of spatial navigation for each paradigm) to be included in the factor analysis. CFA models were run to assess whether unifactorial (cognitive representation), bifactorial (gridded vs. non-gridded and/or RWE vs. VE), and/or trifactorial (paradigm-based and/or task-based) hypotheses would fit the data. Model appropriateness was determined using root-mean-square error of approximation (RMSEA). RMSEA values are less than or equal to 0 and the smaller the better. Model appropriateness was also evaluated using comparative fit index (CFI). CFI values are between 0 and 1 and the larger the better. After identifying the best-fitting model, we added age, KBIT-IQ, and gender to create a sixth model since gender is an individual difference often related to navigation performance (e.g., Coutrot et al., 2019; van der Ham et al., 2020).

We extracted CFA factor scores from the best-fitting model and used them for our final analysis. Each participant had factor scores for each factor in the CFA. Factor scores indicate the participant’s performance relative to other participants for the given factor. We ran linear regression models with the factor scores as the outcome variable and MRT, PTT-A, SBSOD, NSQ, KBIT-IQ, age, and gender as predictors of the navigation behavior. All continuous variables were z-standardized. We ran gvlma (Pena & Slate, 2019) to ensure that each model met the normality assumptions. The Temple Tour model did not satisfy the link function assumption; however, it satisfied all other assumptions and did not demonstrate multicollinearity. We left the model as is for the sake of simplicity and ease of interpretation. We tested the models for multicollinearity of predictors using the “performance” package in R (Lüdecke et al., 2021). All predictors had low correlations as indicated by variance inflation factors below 2, so none were removed from the models (Salmerón et al., 2018).

## Results

Power analysis using semPower in R (Moshagen & Bader, 2023) with a moderate effect size of 0.15, alpha of 0.05, assumed regression slope between latent variables of 0.20, and the smallest degrees of freedom for comparison of factor models (two factors each with three measured variables, df = 8) revealed that a sample size of at least 85 was adequate for 80% power. Power was also calculated using the covariance matrix of the three factors from the optimal model with alpha of 0.05 and beta of 0.20 yielding a minimum sample size of 72. Additional power minimum required sample sizes for each model are included in Table 3.

Descriptive statistics for each paradigm are shown in Table 2. The skewness and kurtosis of the route efficiency tasks for both Square Town and Temple Tour indicated that most participants performed quite well on these tasks. Map building showed different distributions for each paradigm. Square Town map building skewed toward high performance. Temple Tour map building showed a bimodal distribution, with some participants doing quite well and others doing quite poorly. Skewness and kurtosis for Virtual SILCton map building suggested a relatively flat distribution. SILCton between-route pointing had moderate skew and kurtosis. Square Town pointing, SILCton within-route pointing, and Temple Tour pointing had moderate skew and relatively normal kurtosis. SILCton within-route pointing (M = − 26.86, SD = 16.04) was more accurate than SILCton between-route pointing (M = − 55.18, SD = 18.81).

**Table 2:** Descriptive statistics of performance on Virtual SILCton, Square Town, and Temple Tour measures

Measure	M	SD	Min	Max	Skewness	Kurtosis
Square Town route efficiency	− 1.36	0.28	− 2.26	− 0.98	− 1.30	1.63
Square Town map building	0.23	0.25	0.00	0.98	1.65	1.99
Square Town pointing	− 53.12	15.76	− 85.78	− 5.63	0.53	− 0.25
Virtual SILCton map building	0.54	0.27	0.02	0.97	− 0.24	− 1.14
Virtual SILCton within-route pointing	− 26.86	16.04	− 74.56	− 4.25	− 0.66	− 0.24
Virtual SILCton between-route pointing	− 55.18	18.81	− 87.56	− 13.08	0.42	− 0.76
Temple Tour map building	0.46	0.35	0.01	1.00	0.27	− 1.52
Temple Tour pointing	− 30.69	15.02	− 74.80	− 9.20	− 0.65	− 0.45
Temple Tour route efficiency	− 1.22	0.25	− 2.02	− 1.00	− 1.23	0.70

Pointing errors and efficiency error scores are reverse scored (i.e., multiplied by − 1), such that higher values indicate better performance.

### Correlating measures and paradigms

**Fig. 3:**
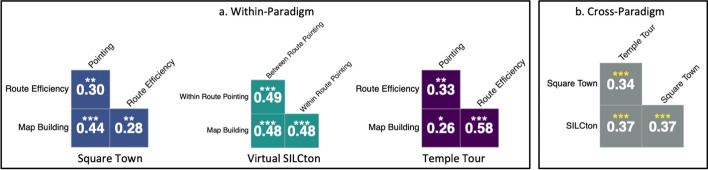
**a** Within-paradigm partial correlations controlling for KBIT-IQ and age. **b** Cross-paradigm partial correlations controlling for KBIT-IQ and age using Z-score indices averages for each paradigm. *** p < 0.001, ** p < 0.01, * p < 0.05

Next, we analyzed the within-paradigm correlations to evaluate internal consistency. We created a partial correlation matrix with p values using performance on each task while controlling for age and KBIT-IQ. Within-paradigm correlations controlling for age and KBIT-IQ were all positive and statistically significant (p < 0.05) as seen in Fig. 3a.

We then evaluated cross-paradigm correlations. We created a partial correlation matrix for z-standardized composite scores controlling for KBIT-IQ and age (Fig. 3b) which yielded positive correlations that were statistically significant (r’s = 0.35–0.38, p’s < 0.001; Fig. 3b).

### Factor models

**Fig. 4:**
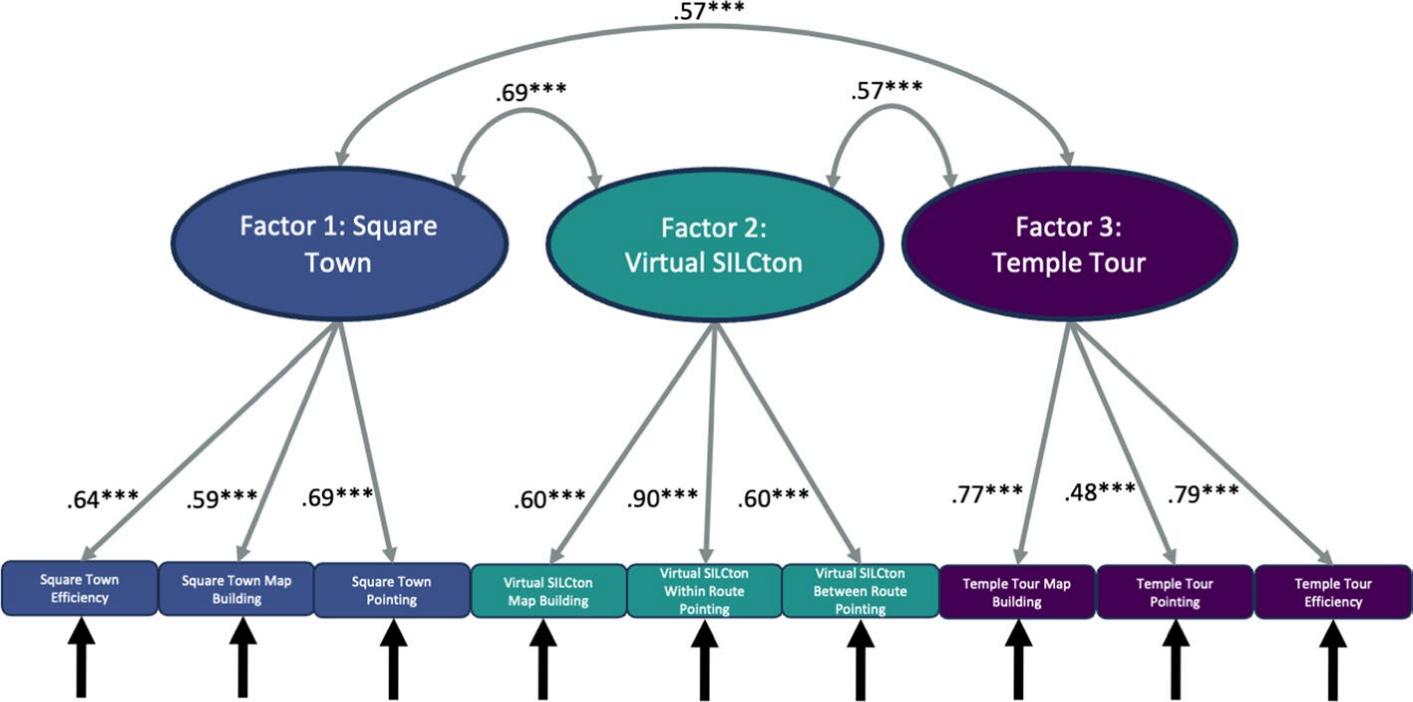
Paradigm-based trifactorial model CFA. This model is the best fit to our data. In this path diagram of the model, latent factors (ovals) are composed of the three observed variables (rectangles) from an individual paradigm. The factors and the variables that reflect them are differentiated by color and number (1, 2, and 3). Arrows between latent factors indicate their correlation and tell us the degree to which they are related. Arrows pointing from the latent factors to the variables indicate the factor loadings. Since factor loadings are standardized, these can also be interpreted like correlations. Black arrows pointing toward the measured variables indicate that there is an error variance term associated with each. *** p < 0.001, ** p < 0.01, * p < 0.05

We found that the data best support the paradigm-based trifactorial model (CFI = 0.94, RMSEA = 0.077, p = 0.039; Fig. 4). In this model, factor 1 consisted of Square Town route efficiency (r = 0.64, p < 0.001), map building (r = 0.59, p < 0.001), and pointing (r = 0.69, p < 0.001). Factor 2 consisted of Virtual SILCton map building (r = 0.60, p < 0.001), within-route pointing (r = 0.60, p < 0.001), and between-route pointing (r = 0.90, p < 0.001). Factor 3 consisted of Temple Tour route efficiency (r = 0.79, p < 0.001), map building (r = 0.77, p < 0.001), and pointing (r = 0.48, p < 0.001). The Temple Tour factor, the RWE, was significantly correlated to the Virtual SILCton factor (r = 0.57, p < 0.001) and the Square Town factor (r = 0.57, p < 0.001). The two VE factors were correlated at r = 0.69 (p < 0.001).

**Fig. 5:**
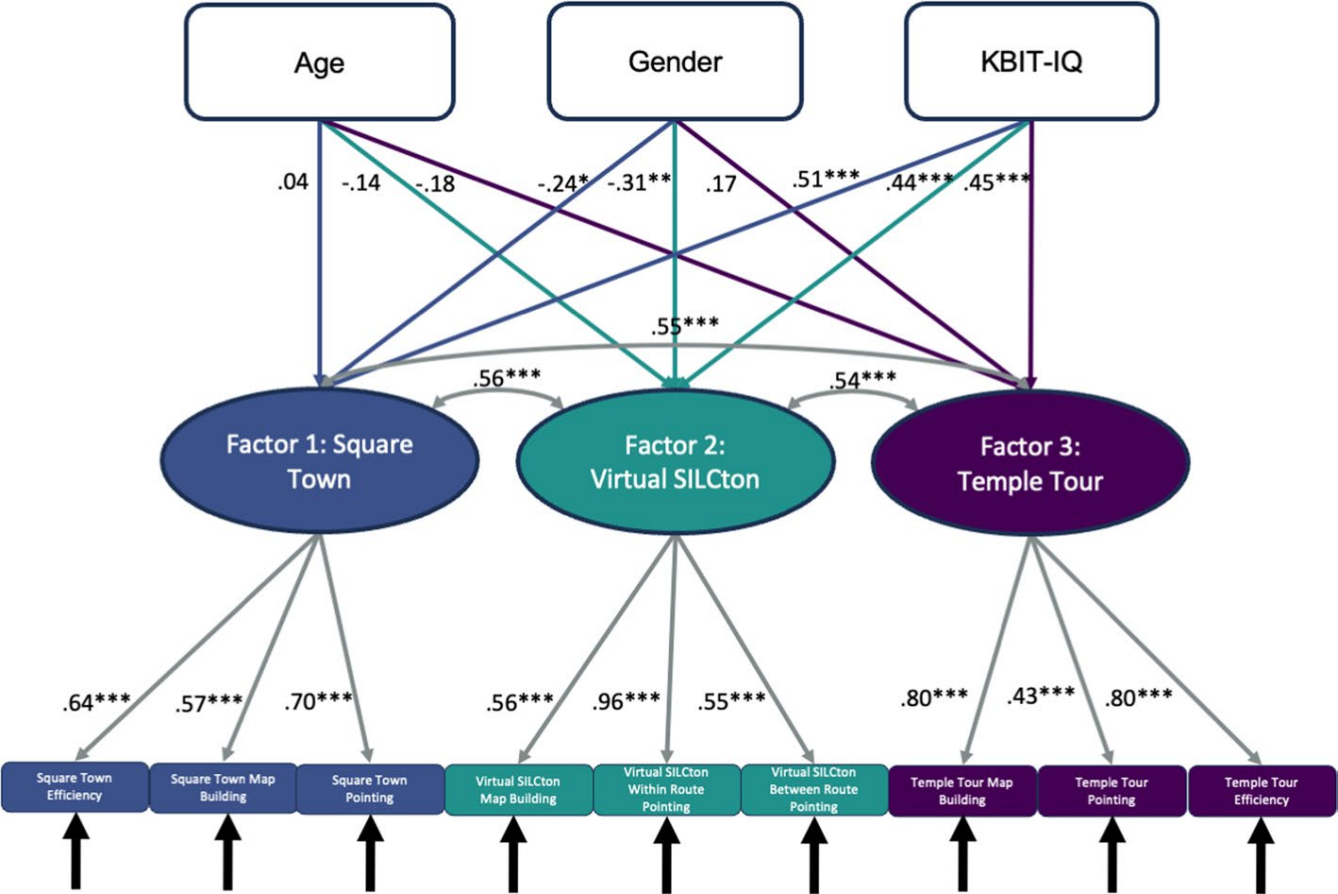
Path diagram for CFA of the optimal model with a layer added for age, gender, and IQ. *** p < 0.001, ** p < 0.01, * p < 0.05

Age, gender, and IQ are important factors in spatial navigation so we built a sixth model (Fig. 5) to include these as variables that may affect the latent factors in the optimal model. We wanted to ensure that the relationships between the factors were not dependent on age, gender, or KBIT-IQ. Age did not correlate with any of the factors. Gender was correlated with performance in the two virtual paradigms. Females tended to perform worse than males on Virtual SILCton and Square Town as indicated by the negative correlations. However, these correlations were relatively weak, Virtual SILCton (r = − 0.24, p < 0.05), and Square Town (r = − 0.31, p < 0.01). KBIT-IQ was strongly correlated with all three factors, Square Town (r = 0.51, p < 0.001), Virtual SILCton (r = 0.44, p < 0.001), and Temple Tour (r = 0.45, p < 0.001). In this model, correlations between factors reduced but did not change significantly as seen in Fig. 5. While the specific values changed for factor loadings, none changed dramatically. This model also showed good fit to our data, with values close to those of the optimal model (CFI = 0.92, RMSEA = 0.078, p = 0.010).

Table 3 shows CFA model statistics for the other hypothesized models. The unifactorial model (CFI = 0.778, RMSEA = 0.142, p < 0.001; Supplemental Fig. 1) represented variance explained by a single dimension, an overarching navigation factor. Each variable significantly contributed to the latent factor (p < 0.001). In the bifactorial model (CFI = 0.853, RMSEA = 0.117, p < 0.001; Supplemental Fig. 2), the variables for Square Town and Temple Tour (i.e., gridded environments) contributed to one factor (factor 1) while those for Virtual SILCton (i.e., non-gridded environment) contributed to another factor (factor 2). Factors 1 and 2 were significantly correlated (r = 0.74, p < 0.001). We ran a bifactorial model again, this time with factor loadings assuming that dimensions represented whether a paradigm used a virtual or real-world environment (CFI = 0.886, RMSEA = 0.104, p = 0.002; Supplemental Fig. 3). The variables all contributed significantly to their respective factors (p < 0.001) and the two factors were significantly correlated (r = 62, p < 0.001). A second trifactorial CFA (Supplemental Fig. 4) with each factor representing a particular task (i.e., route efficiency, map building, and pointing) had an invalid fit (CFI = 0.774, RMSEA = 0.152, p < 0.001), where the covariance matrix of latent variables was not positively definite. Although the literature suggests that these hypothesis-driven organizations of the dataset were all plausible, the models were invalid due to significant CFA RMSEA. In sum, these invalid CFA models support the paradigm-based trifactorial model as best fitting this dataset.

**Table 3:** Fit indices for hypothesis-driven confirmatory factor analysis models

Model	p value	CFI	RMSEA	χ^2^	power required N
unifactorial	< 0.001***	0.778	0.142***	77.83	40 (df = 25)
bifactorial model (VE and RWE)	0.002**	0.886	0.104*	52.24	41 (df = 25)
bifactorial model (gridded and non-gridded build)	< 0.001***	0.853	0.117***	59.72	41 (df = 25)
trifactorial model (task-based)	< 0.001***	0.774	0.152***	75.88	43 (df = 24)
trifactorial optimal model (paradigm-based)	0.039*	0.941	0.077	37.49	43 (df = 24)
trifactorial optimal model with age, gender, and KBIT-IQ	0.010*	0.916	0.078	66.04	31 (df = 42)

*** p < 0.001, ** p < 0.01, * p < 0.05; All power analyses were run with effect size = 0.15, effect measure = RMSEA, alpha = 0.05, and beta = 0.20

### Relating paradigm performance to paradigm-independent spatial measures

Finally, we looked at how MRT, PTT-A, SBSOD, NSQ, age, gender, and KBIT-IQ measures relate to navigation behavior using the trifactorial CFA factor scores. Since the CFA model with age, gender, and IQ also fits the dataset, we generated factor scores from the optimal model shown in Fig. 4. This way, we could directly include age, gender, and IQ in the linear models. Descriptive statistics for the MRT, PTT-A, SBSOD, NSQ, and KBIT-IQ are shown in Table 4. Skewness and kurtosis showed that these measures all had relatively normal distributions, though all but KBIT-IQ were moderately platykurtic.

**Table 4:** Descriptive statistics of performance on Virtual SILCton, Square Town and Temple Tour measures

Measure	M	SD	Min	Max	Skewness	Kurtosis
PTT-A	4.96	1.69	1.05	8.78	− 0.13	− 0.52
MRT	27.96	20.68	− 12.00	80.00	0.25	− 0.71
SBSOD	4.25	1.02	1.80	6.47	− 0.16	− 0.47
NSQ	− 1.23	4.11	− 9.00	8.00	0.28	− 0.79
KBIT-IQ	99.64	11.60	72.00	131.00	0.24	0.15

We ran linear regressions with the factor scores from the optimal CFA model as the outcome variable and MRT, PTT-A, SBSOD, NSQ, Age, Gender, and KBIT-IQ as predictors. We used Cohen’s F_partial_ as an effect size metric with a large effect size as > 0.40, a medium effect size as > 0.25, and a small effect size as > 0.10. We found that MRT was a significant predictor of the factor scores from the VEs, Square Town (β = 0.30, 95% CI (0.09, 0.51), p = 0.006, F_partial_ = 0.51), and Virtual SILCton (β = 0.31, 95% CI (0.10, 0.52), p < 0.005, F_partial_ = 0.52). PTT-A was a significant predictor of the factor scores from Temple Tour (β = 0.30, 95% CI (0.09, 0.50), p = 0.005, F_partial_ = 0.58) and Square Town (β = 0.22, 95% CI (0.04, 0.41), p = 0.023, F_partial_ = 0.62). SBSOD was a significant predictor of factor scores for Virtual SILCton (β = 0.27, 95% CI (0.06, 0.47), p = 0.013, F_partial_ = 0.27) and Temple Tour (β = 0.23, 95% CI (0.01, 0.45), p = 0.044, F_partial_ = 0.15) but with medium and small effect size, respectively. Notably, SBSOD was nearly a significant predictor for Square Town factor score (β = 0.20, 95% CI (0.00, 0.40), p = 0.057, F_partial_ = 0.18). Gender was only a significant predictor of performance in the real-world paradigm, with females outperforming males (β = 0.50, 95% CI (0.08, 0.93), p = 0.022, F_partial_ = 0.27). IQ was a significant predictor of all factor scores, Square Town (β = 0.26, 95% CI (0.07, 0.46), p = 0.010, F_partial_ = 0.28), Virtual SILCton (β = 0.25, 95% CI (0.05, 0.45), p = 0.016, F_partial_ = 0.27), and Temple Tour (β = 0.24, 95% CI (0.03, 0.45), p = 0.030, F_partial_ = 0.24). Table 5 details results.

**Table 5:** Relating MRT, PTT-A, SBSOD, NSQ, and KBIT-IQ to paradigm-based navigation

	Square town factor score		Virtual SILCton factor score		Temple tour factor score	
	Estimate (95% CI)	SE	Estimate (95% CI)	SE	Estimate (95% CI)	SE
Intercept	0.05	0.17	0.13	0.17	− 0.37* (− 0.72, − 0.02)	0.18
PTT-A	0.22* (0.04, 0.41)	0.10	0.17	0.10	0.30** (0.09, 0.50)	0.10
MRT	0.30** (0.09, 0.51)	0.11	0.31** (0.10, 0.52)	0.11	0.21	0.11
NSQ	− 0.13	0.11	− 0.08	0.11	− 0.07	0.11
SBSOD	0.20	0.10	0.27* (0.06, 0.47)	0.11	0.23* (0.01, 0.45)	0.11
Age	0.07	0.09	− 0.04	0.09	− 0.04	0.10
Gender	− 0.07	0.20	− 0.18	0.20	0.50* (0.08, 0.93)	0.22
KBIT-IQ	0.26* (0.07, 0.46)	0.10	0.25* (0.05, 0.45)	0.10	0.24* (0.03, 0.45)	0.11
Adjusted R^2^	0.41		0.39		0.30	

*** p < 0.001; ** p < 0.01; * p < 0.05 model: factor score ~ PTT-A + MRT + NSQ + SBSOD + Age + Gender + KBIT-IQ

## Discussion

This work takes a crucial step toward identifying reliable and valid measures for assessing navigation. Our objective was to investigate the real-world validity of two virtual paradigms, and in doing so establish a model for navigation behavior in young adults. We investigated five possible models based on the existing literature. We found that behavior was correlated across two virtual paradigms, Virtual SILCton and Square Town, and one real-world paradigm, Temple Tour. Since each paradigm had distinctive characteristics apart from different modalities, it was important that we understood where the shared and unique variance in performance stemmed. We tested models factored by environmental build, type of task, and modality to fully understand the characteristics that would isolate correlated behaviors. We determined that specific aspects of the environment (i.e., gridded vs non-gridded), navigation tasks, and modalities (i.e., virtual vs real-world) could not clearly account for differences among paradigms. This result is shown by the significant and high RMSEA values indicating poor model fit. Instead, the optimal model was organized around individual paradigms; each showing unique and shared variance.

Using the factor scores from the optimal model, we showed that performance on each paradigm is differentially predicted by object-based spatial and self-report measures and age, gender, and IQ. At the same time, we identified strong relations between paradigms as also shown in the CFA and partial correlations. Using the optimal model, we constructed a model that included age, gender, and IQ since these factors have previously been shown to influence navigation behavior. We showed good model fit of the paradigm-based trifactorial model with age, gender, and IQ incorporated. These results support the validity of the VE assessments (see also Zhao et al., 2020). It also suggests an underlying pattern of navigation behavior that is relatively invariant across different paradigms. An individual’s navigation behavior is similar across paradigms, suggesting that navigation performance is generalizable and a product of individual cognition. These results have two major implications.

The primary implication of this work is that it is more important to do multi-paradigm testing than just cross-environment, -learning, or -test designs. We should use multiple paradigms because in addition to correlations, we observed paradigm-specific variations in behavior, as shown by the optimal trifactorial model. Future cross-paradigm testing would allow us to confirm that other paradigms are indeed generalizable despite their distinctions, as shown by the paradigms in this study. Paradigm specific differences may be missed without paradigm level testing. Additionally, it is unlikely that any specific aspects of a paradigm such as tasks or “naturalness” can predict a wide distribution of behaviors or cross-situational variability to the same extent. Likewise, as shown by the linear models, single object-based or self-report measures do not always predict performance across paradigms. The object-based and self-report measure results are consistent with the portions of variance in the three-factor model that are not ideal for explaining the overall pattern of within-subject navigation behavior.

These results are consistent with patterns observed in other studies. Topete et al. (under revision) find results consistent with our own, although they do not have a real-world paradigm. Together, our studies suggest that there is unlikely to be a single answer regarding what factors distinguish paradigms. We show that to reach consensus and establish a measure, we should do across-paradigm comparison. Since each paradigm contains a plethora of variation, it is difficult to rely on just one paradigm to generalize individual differences. In this study, we also show that this finding is true between an RWE and two VEs. This result is consistent with Hegarty et al. (2006), who found that RWE vs VE learning is partially dissociated, suggesting that experimenters should be wary of using VEs to measure real-world navigation. Since both our study and Topete et al.’s (under revision) additionally showed a dissociation between VEs, these studies take the former finding a step further and suggest that experimenters should be cautious of using any paradigm to measure performance on another. VE paradigms are approximations of performance in RWE paradigms just as one RWE paradigm approximates performance in another RWE paradigm.

This model makes sense given that different paradigms are often designed for individual experiments and are meant to explain various aspects of navigation. For example, in some paradigms, people behave as if they have access to exact metric information, and in others they behave as though they use the more abstract relational information of the space (Peer et al., 2021, 2024). Testing using only one of these paradigms based on experimental questions might miss other aspects of behavior. We show that there is some specificity of learning in the three environments we used.

One explanation for the degree of specificity shown on the paradigm level is that the combination of different hypothesized factors (e.g., task, environment) adds up to greater explanation of individual differences in navigation than might be expected of the sub-factors on their own. For example, we show that the distinction between virtual versus real-world environment and the distinction between gridded versus non-gridded environment are not enough to explain within-subject variance by themselves. However, both factors are synergistically captured by the paradigm-based model. One way the paradigms can be characterized is as virtual-gridded (Square Town), virtual-non-gridded (Virtual SILCton), and real-world-gridded (Temple Tour).

In line with these findings, we also see that individual differences in performance on mental rotation and perspective taking predicted performance in some paradigms but not others. The factor scores used in the regressions are from the optimal three-factor model. This model depends on both, not just one hypothesized environmental characteristic. The linear models show that PTT-A and MRT predict Square Town factor score, that MRT predicts Virtual SILCton factor score, and that PTT-A predicts Temple Tour factor score. These results show distinctions between paradigms; these distinctions are not sufficient at explaining navigation on their own.

This result supports the idea of using multiple paradigms rather than multiple tests within a paradigm. Since object-based spatial measures predict different factors of navigation behavior on the paradigm level they might indirectly tell us more about its underlying structure. One explanation for mental rotation relating to the VEs but not the RWE can be drawn from a finding by Guo and Song (2023). There is a common transformation mechanism between mental rotation and mapping the magnitude of sensorimotor rotation (Guo & Song, 2023). Specifically, training on visuomotor rotation (VMR) improved speed of mental rotation, and training on mental rotation improved speed of VMR. Navigation in the VEs may have required more mapping between the less familiar controls and the visual motion or location cues on the screen. Thus, MRT performance may have predicted accuracy in the VEs because of the processing required for their VMR aspects. While MRT may be tied to this specific feature of the environment, this feature is just one small distinguishing characteristic of the paradigm. MRT does not provide the “full picture” of within-subject variation in navigation performance that all three factors are sharing.

On the other hand, navigating and pointing in the real world, where bodily mapping of visuomotor rotation is already largely implicit, may rely less on mental rotation. In an environment where viewpoint and surrounding cues are not static on a screen, there may be more flexible encoding of perspective. In a real-world study, perspective taking, but not mental rotation, predicted self-to-object representations (Kozhevnikov et al., 2006). Perspective taking also predicted pointing accuracy (Muffato & Meneghetti, 2020) and mediated the relationship between hippocampus volume and pointing (Schinazi et al., 2013). This relationship is complicated, however, by findings that hippocampal volume in a typical population did not correlate with navigation ability (Weisberg et al., 2019). This nonsignificant correlation could be composed of a mix of significant and nonsignificant correlations among high versus low spatial-ability subgroups of individuals (He & Brown, 2019).

It is also possible that perspective taking in particular, not general navigation ability, is correlated with hippocampal volume. This point could explain why perspective taking predicts navigation performance in the Square Town and the Temple Tour paradigms but not in the Virtual SILCton paradigm. Consistent with previous work (Fields & Shelton, 2006; Münzer et al., Münzer et al., 2020; Nazareth et al., 2018), we show that perspective taking tasks are not uniquely related to real-world over virtual paradigms. Again, supporting the idea that navigation may better be explained at the paradigm level. Additionally, since previous studies that found correlations between perspective taking and navigation tasks did not always use gridded environments (e.g., Schinazi et al., 2013), this environmental structure is unlikely to explain the common predictor between Square Town and Temple Tour. Instead, the paradigms that are predicted by perspective taking both include route efficiency tasks, where movements are less constrained. The additional variations in viewpoints may influence the greater use of perspectives to support spatial memory.

Gender also relates differently across paradigms. In the CFA model, gender correlates with the VEs, but in the linear models, gender only relates to performance in the RWE. Males tend to outperform females in the virtual paradigms, but females did better in the RWE. This pattern may have to do with gender differences in navigation that stem from differences in strategy, motivation, or stress (Schinazi et al., 2023).

There could still be other paradigm-distinguishing characteristics that are not captured by our optimal model. For this reason, we are hesitant to make the claim that only the characteristics we show in this study are what distinguishes navigation paradigms. At the paradigm level, there are a few factors that are unique to the paradigms we used here and could be contributing to their orthogonal portions. The method of encoding could be one contributor. We sought to make comparisons in a way that was representative of the range of paradigms in the literature. Sometimes paradigm encoding happens on one day and then retrieval on another, as was the case for Square Town. In other cases, there might be a social component to encoding as in Temple Tour. Other times, navigational encoding may be self-paced, like in Virtual SILCton. This potential experimental decision could contribute to the distinction between paradigms. Scale could also have an influence as all three paradigms used environments that varied in size. Future cross-paradigm testing may help reveal important characteristics that limit the generalizability of paradigms on individual differences in navigation.

Prior work shows that individual studies can potentially be somewhat limited in generality (e.g., Ekstrom & Hill, 2023). We show that this variance can be assessed by paradigm level differences instead of individual features such as task or modality. Hence, studies should use multiple paradigms. However, it is unclear just how many paradigms are sufficient for a good description of navigation behavior. Rekers et al. (2025) provided a good example of the reliability and validity of a single paradigm. Since our results also show differences in how MRT and PTT-A predict performance, this study supports doing meta-analysis of what past research has found from including these measures.

The second implication of this work is that none of the existing paradigms used in this study should be scrapped due to lack of validity. By doing the cross-paradigm comparison (as opposed to say, cross-task comparison), we know that all the paradigms used in the current study strongly correlate with each other. This demonstrates real-world validity for the virtual paradigms, Virtual SILCton and Square Town (though each with some unique variance). There is a large portion of commonality in learning for the three environments. The correlated variance is poorly explained by any single paradigm characteristic used here. This result is supported by the poor fit of the other CFA models. By doing cross-paradigm comparisons, researchers can ensure the validity of their work and clarify what aspects of their paradigms are specific rather than generalizable. Since every paradigm-factor strongly correlates with each other, we suggest that the shared variance in the optimal model is tied to a unitary cognitive component that each paradigm is tapping into.

This dimension may also relate to another interesting aspect of our data, that both self-report measures used were poor predictors of navigation performance. While the SBSOD did show some significance, its effect size was relatively small. Here, we show that failure of self-report measures is not due to movement or body-based cues, as suggested elsewhere for similar findings (Garg et al., 2024). The NSQ does not predict performance in any environment. The SBSOD predicts performance on both the VE and RWE. They may both be poor at explaining the same trait instead of individual characteristics of paradigms. One possibility is that the self-reports assess states rather than measuring individual cognitive differences (traits). Heth et al. (2002) found that the SBSOD was only correlated with navigation performance when completed after but not before, suggesting that it may reflect a subset of recent events rather than representing individual’s traits. However, in our study the SBSOD was completed before any of the navigational paradigms. Another unexplored possibility is that over time these measures have lost their reliability.

### Limitations and future directions

We note limitations due to sample, study demands, and tasks. We see these limitations as avenues for future research. In the present study, our findings only refer to a young adult sample. As expected, there are no significant statistical relationships with age in the CFA or linear models because all participants are from the same age group. Future research should investigate our findings across different stages of development, such as into later adulthood.

Additionally, we used a fixed order of paradigms because there may be variance associated with using a balanced order (e.g., participant fatigue) that we were not interested in for this study. While a counterbalanced order could avoid participants being influenced by earlier paradigms or fatigue, fixed order ensures that if there is paradigm-to-paradigm influence it is held constant across subjects and controlled for in the design. The fixed and counterbalanced orders are useful in different ways. A future study using a counterbalanced order might be interested in which of the paradigms is most influential, but this is not what the current study asks. This study cannot address which factor(s) or paradigm(s) would make the best measures, but instead focused on modeling the patterns of variance within subjects to quantify individual differences in navigation. Likewise, balanced order was not important for addressing individual differences using the MRT and PTT-A. Splitting MRT and PTT-A up onto two separate days prevented either session from being too long. An interest of the present study is to shorten the time demands of navigation research.

“Confirmatory factor analysis” can be a misleading term, because this work is still in its early stages. The present study corroborates hypothesized models based on the existing literature. Structural equation modeling (SEM) is the ideal way to get the full picture of this work. However, there needs to be substantially more data to fully interrogate the structure of behavior performance across these tasks. Given the resources it would take to collect this sample, we do not see this work as being feasible for most labs doing navigational research. The primary issue is with the number of measures and the length of each measure. This limitation is one of the reasons we ran the current study, as it is the preliminary step in streamlining the measures necessary to allow eventual SEM.

A last important point related to study demand limitations is that there are other reliability and validity tests that are not addressed by the present research and are worth following up on in future investigations. Divergent validity could be evaluated in future work but was not relevant to this study. We used tasks that are repeatedly used in the literature and control for IQ in our models because it could be a confounding factor. IQ showed significance in our models but did not significantly alter the optimal CFA model. Another is within-subject test–retest reliability, which would have been difficult to collect given the time requirements for participants and resources necessary for equitable compensation. However, our inclusion of Virtual SILCton provides additional evidence that our novel experiment is reliable. Virtual SILCton has been used repeatedly across different samples (Brucato et al., 2022; Nazareth et al., 2018; Weisberg et al., 2014; Weisberg & Newcombe, 2016; Zhao et al., 2020) with reliable correlation between within-route and between-route pointing ranging from r = 0.45 (Weisberg & Newcombe, 2016) to r = 0.55 (Weisberg et al., 2014). This paradigm is a reliable measure of spatial integration but does not say much about its real-world validity. Our own results are consistent with these past findings (r = 0.49) so that Virtual SILCton provides another source of support for the reliability of the present findings.

We did not include a task for route efficiency in our version of the Virtual SILCton paradigm. Topete et al. (under review) added a route efficiency test to the Virtual SILCton paradigm and showed that this measure correlates with the map building and pointing tasks. Our within-paradigm correlations for the other Virtual SILCton measures are consistent with Topete et al., suggesting that this specific measure is unlikely to be the source of failure for the other models.

Normality of variables is a requirement of CFA because it is a multivariate test. We found high skew for some of the variables, particularly for the route efficiency tests where participants tended to do quite well. Log transforming variables that exceeded ± 0.5 in skewness for the CFA did not help meet this assumption. This violation is an important limitation of our results that would likely need a more difficult route efficiency task to fix.

There are also other inputs to the navigation system that we do not account for in our tasks such as metrics of sensorimotor, vestibular, and proprioceptive cues (Steel et al., 2021). Considering the results from the current study which demonstrate paradigm-level individual differences, cross-paradigm testing is likely the best way to address the large number of inputs to the navigation system. We hope that this work will allow greater efficiency in future studies, allowing more work investigating these open questions.

## Conclusions

To conclude, there are several implications of this study for the reliable and valid measurement of navigation. The literature says that rodents have consistent ways of mapping the world, based on data collected via intracranial measurement (Hafting et al., 2005; McNaughton et al., 1983; Moser et al., 2008; Muller et al., 1987; O'Keefe & Dostrovsky, 1971; O'Keefe & Speakman, 1987; Wilson & McNaughton, 1993). Our study shows that human navigation is also likely based on a single cognitive dimension of the individual. Navigation behavior at the paradigm level seems to have generalizable results. The pattern of this cognition and why it is presented in certain ways in different situations remains an open question. Of immediate practical relevance, this study suggests that none of the existing paradigms used in this study should be scrapped due to lack of validity. The patterns in this agreement imply that understanding navigation behavior is not necessarily a matter of increasing measurement through the number of tasks. Instead, more than one paradigm is more important to reach reliable and valid measurement (consensus) and understand nuanced individual differences in navigation behavior.

## Supplementary Information

Supplementary Material 1.

## Data Availability

The data are available on OSF (https://osf.io/xcyks/?view_only=6af4ac6afb784a359db2b950ae077b72). The scripts written for the cleaning and analyses are available on the Github repository managed by the corresponding author (https://github.com/JacobLader/Reliable_and_valid_navigation_testing). None of the experiments were preregistered.

## Abbreviations

RWE: Real-world environment
VE: Virtual environment
SBSOD: Santa Barbara sense of direction scale
MRT: Mental rotation test
Virtual SILCton: Spatial intelligence learning center test of navigation
KBIT-IQ: Kaufman brief intelligence test 2nd edition
NSQ: Navigation strategy questionnaire
PTT-A: Perspective taking test—adults
CFA: Confirmatory factor analysis
VMR: Visuomotor rotation
RMSEA: Root-mean-square error of approximation
CFI: Comparative fit index

## References

[CR1] Allen, G. L., Kirasic, K. C., Dobson, S. H., Long, R. G., & Beck, S. (1996). Predicting environmental learning from spatial abilities: An indirect route. *Intelligence,**22*(3), 327–355. 10.1016/s0160-2896(96)90026-4

[CR2] Allison, S. L., Fagan, A. M., Morris, J. C., & Head, D. (2016). Spatial navigation in preclinical Alzheimer’s disease. *Journal of Alzheimer’s Disease: JAD,**52*(1), 77–90. 10.3233/JAD-150855 26967209 10.3233/JAD-150855 PMC5091813

[CR3] Blajenkova, O., Motes, M. A., & Kozhevnikov, M. (2005). Individual differences in the representations of novel environments. *Journal of Environmental Psychology,**25*(1), 97–109. 10.1016/j.jenvp.2004.12.003

[CR4] Brucato, M., Frick, A., Pichelmann, S., Nazareth, A., & Newcombe, N. S. (2023). Measuring spatial perspective taking: Analysis of four measures using item response theory. *Topics in Cognitive Science,**15*(1), 46–74. 10.1111/tops.12597 35032360 10.1111/tops.12597

[CR5] Brucato, M., Nazareth, A., & Newcombe, N. S. (2022). Longitudinal development of cognitive mapping from childhood to adolescence. *Journal of Experimental Child Psychology,**219*, 105412. 10.1016/j.jecp.2022.105412 35272067 10.1016/j.jecp.2022.105412

[CR6] Brunec, I. K., Bellana, B., Ozubko, J. D., Man, V., Robin, J., Liu, Z. X., Grady, C., Rosenbaum, R. S., Winocur, G., Barense, M. D., & Moscovitch, M. (2018). Multiple scales of representation along the hippocampal anteroposterior axis in humans. *Current Biology,**28*(13), 2129-2135.e6. 10.1016/j.cub.2018.05.016 29937352 10.1016/j.cub.2018.05.016

[CR7] Carbon, C.-C. (2013). BiDimRegression: Bidimensional regression modeling using R. *Journal of Statistical Software Code Snippets,**52*(1), 1–11. 10.18637/jss.v052.c01

[CR8] Chrastil, E. R., & Warren, W. H. (2014). From cognitive maps to cognitive graphs. *PLoS ONE,**9*(11), e112544. 10.1371/journal.pone.0112544 25389769 10.1371/journal.pone.0112544 PMC4229194

[CR9] Cortes, R. A., Peterson, E. G., Kraemer, D. J. M., Kolvoord, R. A., Uttal, D. H., Dinh, N., Weinberger, A. B., Daker, R. J., Lyons, I. M., Goldman, D., & Green, A. E. (2022). Transfer from spatial education to verbal reasoning and prediction of transfer from learning-related neural change. *Science Advances,**8*(32), eabo3555. 10.1126/sciadv.abo3555 35947663 10.1126/sciadv.abo3555 PMC9365289

[CR10] Coutrot, A., Schmidt, S., Coutrot, L., Pittman, J., Hong, L., Wiener, J. M., Hölscher, C., Dalton, R. C., Hornberger, M., & Spiers, H. J. (2019). Virtual navigation tested on a mobile app is predictive of real-world wayfinding navigation performance. *PLoS ONE*. 10.1371/journal.pone.0213272 30883560 10.1371/journal.pone.0213272 PMC6422266

[CR11] Diamond, N. B., Abdi, H., & Levine, B. (2020). Different patterns of recollection for matched real-world and laboratory-based episodes in younger and older adults. *Cognition,**202*, 104309. 10.1016/j.cognition.2020.104309 32388006 10.1016/j.cognition.2020.104309

[CR12] Doner, S., Zheng, J., McAvan, A. S., Starrett, M. J., Campbell, H., Sanders, D., & Ekstrom, A. (2023). Evidence for flexible navigation strategies during spatial learning involving path choices. *Spatial Cognition & Computation,**23*(3), 233–262. 10.1080/13875868.2022.2158090

[CR13] Ekstrom, A. D., & Hill, P. F. (2023). Spatial navigation and memory: A review of the similarities and differences relevant to brain models and age. *Neuron,**111*(7), 1037–1049. 10.1016/j.neuron.2023.03.001 37023709 10.1016/j.neuron.2023.03.001 PMC10083890

[CR14] Ericson, J. D., & Warren, W. H. (2020). Probing the invariant structure of spatial knowledge: Support for the cognitive graph hypothesis. *Cognition,**200*, 104276. 10.1016/j.cognition.2020.104276 32450417 10.1016/j.cognition.2020.104276

[CR15] Fields, A. W., & Shelton, A. L. (2006). Individual skill differences and large-scale environmental learning. *Journal of Experimental Psychology: Learning, Memory, and Cognition,**32*(3), 506–515. 10.1037/0278-7393.32.3.506 16719662 10.1037/0278-7393.32.3.506

[CR17] Frick, A., Möhring, W., & Newcombe, N. S. (2014). Picturing perspectives: Development of perspective-taking abilities in 4- to 8-year-olds. *Frontiers in Psychology,**5*, 386. 10.3389/fpsyg.2014.00386 24817860 10.3389/fpsyg.2014.00386 PMC4012199

[CR18] Friedman, A., & Kohler, B. (2003). Bidimensional regression: Assessing the configural similarity and accuracy of cognitive maps and other two-dimensional data sets. *Psychological Methods,**8*(4), 468–491. 10.1037/1082-989X.8.4.468 14664683 10.1037/1082-989X.8.4.468

[CR19] Galea, L. A. M., & Kimura, D. (1993). Sex differences in route-learning. *Personality and Individual Differences,**14*(1), 53–65. 10.1016/0191-8869(93)90174-2

[CR20] Garg, T., Velasco, P. F., Patai, E. Z., Malcolm, C. P., Kovalets, V., Bohbot, V. D., Coutrot, A., Hegarty, M., Hornberger, M., & Spiers, H. J. (2024). The relationship between object-based spatial ability and virtual navigation performance. *PLoS ONE,**19*(5), e0298116. 10.1371/journal.pone.0298116 38722850 10.1371/journal.pone.0298116 PMC11081363

[CR21] Golledge, R. G. (1999). *Wayfinding behavior: Cognitive mapping and other spatial processes*. John Hopkins.

[CR22] Goodroe, S., Fernandez Velasco, P., Gahnstrom, C. J., Wiener, J., Coutrot, A., Hornberger, M., & Spiers, H. J. (2024). Predicting real-world navigation performance from a virtual navigation task in older adults. 10.1101/2024.07.23.604766 10.1371/journal.pone.0317026 PMC11771902 39869655

[CR23] Guo, J., & Song, J. H. (2023). Reciprocal facilitation between mental and visuomotor rotations. *Scientific Reports,**13*(1), 825. 10.1038/s41598-022-26397-3 36646722 10.1038/s41598-022-26397-3 PMC9842739

[CR24] Hafting, T., Fyhn, M., Molden, S., Moser, M. B., & Moser, E. I. (2005). Microstructure of a spatial map in the entorhinal cortex. *Nature,**436*(7052), 801–806. 10.1038/nature03721 15965463 10.1038/nature03721

[CR25] Hegarty, M., Montello, D. R., Richardson, A. E., Ishikawa, T., & Lovelace, K. (2006). Spatial abilities at different scales: Individual differences in aptitude-test performance and spatial-layout learning. *Intelligence,**34*(2), 151–176. 10.1016/j.intell.2005.09.005

[CR26] Hegarty, M., Richardson, A. E., Montello, D. R., Lovelace, K., & Subbiah, I. (2002). Development of a self-report measure of environmental spatial ability. *Intelligence,**30*(5), 425–447. 10.1016/S0160-2896(02)00116-2

[CR27] Hegarty, M., & Waller, D. (2004). A dissociation between mental rotation and perspective-taking spatial abilities. *Intelligence,**32*, 175–191. 10.1016/j.intell.2003.12.001

[CR28] Hegarty, M., & Waller, D. A. (2005). Individual differences in spatial abilities. In P. Shah & A. Miyake (Eds.), *The Cambridge handbook of visuospatial thinking* (pp. 121–169). Cambridge University Press. 10.1017/CBO9780511610448.005

[CR29] Hejtmanek, L., Starrett, M., Ferrer, E., & Ekstrom, A. D. (2020). How much of what we learn in virtual reality transfers to real-world navigation? *Multisensory Research,**33*(4–5), 479–503. 10.1163/22134808-20201445 31972540 10.1163/22134808-20201445

[CR30] He, Q., & Brown, T. I. (2019). Environmental barriers disrupt grid-like representations in humans during navigation. *Current Biology,**29*(16), 2718-2722.e3. 10.1016/j.cub.2019.06.072 31378608 10.1016/j.cub.2019.06.072 PMC7123550

[CR32] Heth, C. D., Cornell, E. H., & Flood, T. L. (2002). Self-ratings of sense of direction and route reversal performance. *Applied Cognitive Psychology,**16*, 309–324. 10.1002/acp.795

[CR33] Ishikawa, T., & Montello, D. R. (2006). Spatial knowledge acquisition from direct experience in the environment: Individual differences in the development of metric knowledge and the integration of separately learned places. *Cognitive Psychology,**52*(2), 93–129. 10.1016/j.cogpsych.2005.08.003 16375882 10.1016/j.cogpsych.2005.08.003

[CR34] Kalantari, S., Mostafavi, A., Xu, B. T., Lee, A. S., & Yang, Q. (2024). Comparing spatial navigation in a virtual environment vs an identical real environment across the adult lifespan. *Computers in Human Behavior,**157*, 108210. 10.1016/j.chb.2024.108210

[CR35] Kaufman, A. S., & Kaufman, N. L. (2004). *Kaufman brief intelligence test kbit 2; manual* (2. ed.). Pearson.

[CR36] Kozlowski, L. T., & Bryant, K. J. (1977). Sense of direction, spatial orientation, and cognitive maps. *Journal of Experimental Psychology: Human Perception and Performance,**3*(4), 590–598. 10.1037/0096-1523.3.4.590

[CR37] Kozhevnikov, M., Motes, M. A., Rasch, B., & Blajenkova, O. (2006). Perspective-taking versus mental rotation transformations and how they predict spatial navigation performance. *Applied Cognitive Psychology,**20*, 397–417. 10.1002/acp.1192

[CR38] Lüdecke, D., Ben-Shachar, M. S., Patil, I., Waggoner, P., & Makowski, D. (2021). Performance: An R package for assessment, comparison and testing of statistical models. *Journal of Open Source Software,**6*(60), 3139. 10.21105/joss.03139

[CR40] McNaughton, B. L., Barnes, C. A., & O’Keefe, J. (1983). The contributions of position, direction, and velocity to single unit activity in the hippocampus of freely-moving rats. *Experimental Brain Research,**52*, 41–49. 10.1007/BF00237147 6628596 10.1007/BF00237147

[CR41] Meneghetti, C., Miola, L., Toffalini, E., Pastore, M., & Pazzaglia, F. (2021). Learning from navigation, and tasks assessing its accuracy: The role of visuospatial abilities and wayfinding inclinations. *Journal of Environmental Psychology,**75*, 101614. 10.1016/j.jenvp.2021.101614

[CR42] Moeser, S. D. (1988). Cognitive mapping in a complex building. *Environment and Behavior,**20*(1), 21–49. 10.1177/0013916588201002

[CR43] Moffat, S. D. (2009). Aging and spatial navigation: What do we know and where do we go? *Neuropsychology Review,**19*(4), 478–489. 10.1007/s11065-009-9120-3 19936933 10.1007/s11065-009-9120-3

[CR44] Moffat, S. D., Hampson, E., & Hatzipantelis, M. (1998). Navigation in a “virtual” maze: Sex differences and correlation with psychometric measures of spatial ability in humans. *Evolution and Human Behavior,**19*(2), 73–87. 10.1016/s1090-5138(97)00104-9

[CR45] Montello, D. R. (2005). Navigation. In P. Shah & A. Miyake (Eds.), *The Cambridge handbook of visuospatial thinking* (pp. 257–294). Cambridge University Press.

[CR46] Moser, E. I., Kropff, E., & Moser, M. B. (2008). Place cells, grid cells, and the brain’s spatial representation system. *Annual Review of Neuroscience,**31*, 69–89. 10.1146/annurev.neuro.31.061307.090723 18284371 10.1146/annurev.neuro.31.061307.090723

[CR47] Moshagen, M., & Bader, M. (2023). semPower: Power analyses for SEM. R package version 2.1.0. https://CRAN.R-project.org/package=semPower

[CR48] Muffato, V., & Meneghetti, C. (2020). Learning a path from real navigation: The advantage of initial view, cardinal North and visuo-spatial ability. *Brain Sciences,**10*(4), 204. 10.3390/brainsci10040204 32244674 10.3390/brainsci10040204 PMC7226432

[CR49] Muffato, V., Meneghetti, C., & De Beni, R. (2020). The role of visuo-spatial abilities in environment learning from maps and navigation over the adult lifespan. *British Journal of Psychology,**111*, 70–91. 10.1111/bjop.12384 30927263 10.1111/bjop.12384

[CR500] Muffato, V., Borella, E., Pazzaglia, F., & Meneghetti, C. (2022). Orientation Experiences and Navigation Aid Use: A Self-Report Lifespan Study on the Role of Age and Visuospatial Factors. *International Journal of Environmental Research and Public Health, 19*(3), 1225. 10.3390/ijerph19031225 35162250 10.3390/ijerph19031225 PMC8835153

[CR51] Muller, R. U., Kubie, J. L., & Ranck, J. B., Jr. (1987). Spatial firing patterns of hippocampal complex-spike cells in a fixed environment. *The Journal of Neuroscience: THe Official Journal of the Society for Neuroscience,**7*(7), 1935–1950. 10.1523/JNEUROSCI.07-07-01935.1987 3612225 10.1523/JNEUROSCI.07-07-01935.1987 PMC6568929

[CR52] Münzer, S., Lörch, L., & Frankenstein, J. (2020). Wayfinding and acquisition of spatial knowledge with navigation assistance. *Journal of Experimental Psychology: Applied,**26*(1), 73–88. 10.1037/xap0000237 31246054 10.1037/xap0000237

[CR53] Muryy, A., & Glennerster, A. (2018). Pointing errors in non-metric virtual environments. *Lecture Notes in Computer Science*. 10.1007/978-3-319-96385-3_4

[CR54] Nazareth, A., Weisberg, S. M., Margulis, K., & Newcombe, N. S. (2018). Charting the development of cognitive mapping. *Journal of Experimental Child Psychology,**170*, 86–106. 10.1016/j.jecp.2018.01.009 29453130 10.1016/j.jecp.2018.01.009

[CR55] Newcombe, N. S., Hegarty, M., & Uttal, D. (2023). Building a cognitive science of human variation: Individual differences in spatial navigation. *Topics in Cognitive Science,**15*, 6–14. 10.1111/tops.12626 36203368 10.1111/tops.12626

[CR56] O’Keefe, J., & Dostrovsky, J. (1971). The hippocampus as a spatial map. Preliminary evidence from unit activity in the freely-moving rat. *Brain Research,**34*(1), 171–175. 10.1016/0006-8993(71)90358-1 5124915 10.1016/0006-8993(71)90358-1

[CR58] O’Keefe, J., & Speakman, A. (1987). Single unit activity in the rat hippocampus during a spatial memory task. *Experimental Brain Research,**68*(1), 1–27. 10.1007/BF00255230 3691688 10.1007/BF00255230

[CR501] Pazzaglia, F., & De Beni, R. (2001). Strategies of processing spatial information in survey and landmarkcentred individuals. *European Journal of Cognitive Psychology, 13*(4), 493–508. 10.1080/09541440042000124

[CR59] Pazzaglia, F., & De Beni, R. (2006). Are people with high and low mental rotation abilities differently susceptible to the alignment effect? *Perception,**35*(3), 369–383. 10.1068/p5465 16619952 10.1068/p5465

[CR60] Peer, M., Brunec, I. K., Newcombe, N. S., & Epstein, R. A. (2021). Structuring knowledge with cognitive maps and cognitive graphs. *Trends in Cognitive Sciences,**25*(1), 37–54. 10.1016/j.tics.2020.10.004 33248898 10.1016/j.tics.2020.10.004 PMC7746605

[CR61] Peer, M., Nadar, C., & Epstein, R. A. (2024). The format of the cognitive map depends on the structure of the environment. *Journal of Experimental Psychology: General,**153*(1), 224–240. 10.1037/xge0001498 37843528 10.1037/xge0001498 PMC10872840

[CR62] Peirce, J., Gray, J. R., Simpson, S., MacAskill, M., Höchenberger, R., Sogo, H., Kastman, E., & Lindeløv, J. K. (2019). PsychoPy2: Experiments in behavior made easy. *Behavior Research Methods,**51*(1), 195–203. 10.3758/s13428-018-01193-y 30734206 10.3758/s13428-018-01193-y PMC6420413

[CR63] Pena, E. A., & Slate, E. H. (2019). _gvlma: Global validation of linear models assumptions_. R package version 1.0.0.3. https://CRAN.R-project.org/package=gvlma

[CR64] Peters, M., Laeng, B., Latham, K., Jackson, M., Zaiyouna, R., & Richardson, C. (1995). A redrawn Vandenberg and Kuse mental rotations test: Different versions and factors that affect performance. *Brain and Cognition,**28*(1), 39–58. 10.1006/brcg.1995.1032 7546667 10.1006/brcg.1995.1032

[CR65] R Core Team. (2024). _R: A language and environment for statistical computing_. R Foundation for Statistical Computing, Vienna, Austria. https://www.R-project.org/

[CR66] Rekers, S., Meyer, T. C., & Finke, C. (2025). Psychometric data and regression-based norms for the Virtual Environments Navigation Assessment for young and middle-aged adults (VIENNA Young). 10.31234/osf.io/h56bd

[CR67] Rosseel, Y. (2012). lavaan: An R package for structural equation modeling. *Journal of Statistical Software,**48*(2), 1–36. 10.18637/jss.v048.i02

[CR68] Salmerón, R., García, C. B., & García, J. (2018). Variance inflation factor and condition number in multiple linear regression. *Journal of Statistical Computation and Simulation,**88*(12), 2365–2384. 10.1080/00949655.2018.1463376

[CR70] Saucier, D. M., Green, S. M., Leason, J., MacFadden, A., Bell, S., & Elias, L. J. (2002). Are sex differences in navigation caused by sexually dimorphic strategies or by differences in the ability to use the strategies? *Behavioral Neuroscience,**116*(3), 403–410. 10.1037/0735-7044.116.3.403 12049321 10.1037//0735-7044.116.3.403

[CR71] Schinazi, V. R., Nardi, D., Newcombe, N. S., Shipley, T. F., & Epstein, R. A. (2013). Hippocampal size predicts rapid learning of a cognitive map in humans. *Hippocampus,**23*(6), 515–528. 10.1002/hipo.22111 23505031 10.1002/hipo.22111 PMC3690629

[CR72] Schinazi, V. R., Meloni, D., Grübel, J., Angus, D. J., Baumann, O., Weibel, R. P., Jeszenszky, P., Hölscher, C., & Thrash, T. (2023). Motivation moderates gender differences in navigation performance. *Scientific Reports,**13*(1), 15995. 10.1038/s41598-023-43241-4 37749312 10.1038/s41598-023-43241-4 PMC10520045

[CR73] Sholl, M. J. (1988). The relationship between sense of direction and mental geographic updating. *Intelligence,**12*(3), 299–314. 10.1016/0160-2896(88)90028-1

[CR74] Siegel, A. W., & White, S. H. (1975). The development of spatial representations of large-scale environments. *Advances in Child Development and Behavior*. 10.1016/s0065-2407(08)60007-5 1101663 10.1016/s0065-2407(08)60007-5

[CR75] Steel, A., Robertson, C. E., & Taube, J. S. (2021). Current promises and limitations of combined virtual reality and functional magnetic resonance imaging research in humans: A commentary on Huffman and Ekstrom (2019). *Journal of Cognitive Neuroscience,**33*(2), 159–166. 10.1162/jocn_a_01635 33054553 10.1162/jocn_a_01635 PMC10284033

[CR76] Tobler, W. R. (1994). Bidimensional regression. *Geographical Analysis,**26*, 187–212. 10.1111/j.1538-4632.1994.tb00320.x

[CR77] Topete, A., He, C., & Hegarty, M. (under review). Navigation ability in different environment types: Does context matter?

[CR78] Vandenberg, S. G., & Kuse, A. R. (1978). Mental rotations, a group test of three-dimensional spatial visualization. *Perceptual and Motor Skills,**47*(2), 599–604. 10.2466/pms.1978.47.2.599 724398 10.2466/pms.1978.47.2.599

[CR79] van der Ham, I. J. M., & Claessen, M. H. G. (2020). How age relates to spatial navigation performance: Functional and methodological considerations. *Ageing Research Reviews,**58*, 101020. 10.1016/j.arr.2020.101020 31954190 10.1016/j.arr.2020.101020

[CR80] van der Ham, I. J. M., Claessen, M. H. G., Evers, A. W. M., & van der Kuil, M. N. A. (2020). Large-scale assessment of human navigation ability across the lifespan. *Scientific Reports,**10*(1), 3299. 10.1038/s41598-020-60302-0 32094394 10.1038/s41598-020-60302-0 PMC7039892

[CR503] Weisberg, S. M., & Newcombe, N. S. (2016). How do (some) people make a cognitive map? Routes, places, and working memory. *Journal of Experimental Psychology. Learning, Memory, and Cognition, 42*(5), 768–785. 10.1037/xlm0000200 10.1037/xlm0000200 26595065

[CR83] Weisberg, S. M., & Newcombe, N. S. (2018). Cognitive maps: Some people make them, some people struggle. *Current Directions in Psychological Science,**27*(4), 220–226. 10.1177/0963721417744521 30122809 10.1177/0963721417744521 PMC6095672

[CR84] Weisberg, S. M., Newcombe, N. S., & Chatterjee, A. (2019). Everyday taxi drivers: Do better navigators have larger hippocampi? *Cortex: A Journal Devoted to the Study of the Nervous System and Behavior,**115*, 280–293. 10.1016/j.cortex.2018.12.024 30884282 10.1016/j.cortex.2018.12.024 PMC6513697

[CR85] Weisberg, S. M., Schinazi, V. R., Newcombe, N. S., Shipley, T. F., & Epstein, R. A. (2014). Variations in cognitive maps: Understanding individual differences in navigation. *Journal of Experimental Psychology, Learning, Memory, and Cognition,**40*(3), 669–682. 10.1037/a0035261 24364725 10.1037/a0035261

[CR86] Wiener, J. M., Büchner, S. J., & Hölscher, C. (2009). Taxonomy of human wayfinding tasks: A knowledge-based approach. *Spatial Cognition & Computation,**9*(2), 152–165. 10.1080/13875860902906496

[CR87] Wilson, M. A., & McNaughton, B. L. (1993). Dynamics of the hippocampal ensemble code for space. *Science,**261*(5124), 1055–1058. 10.1126/science.8351520 8351520 10.1126/science.8351520

[CR88] Wolbers, T., & Hegarty, M. (2010). What determines our navigational abilities? *Trends in Cognitive Sciences,**14*(3), 138–146. 10.1016/j.tics.2010.01.001 20138795 10.1016/j.tics.2010.01.001

[CR90] Zetzsche, C., Wolter, J., Galbraith, C., & Schill, K. (2009). Representation of space: Image-like or sensorimotor? *Spatial Vision,**22*(5), 409–424. 10.1163/156856809789476074 19814904 10.1163/156856809789476074

[CR91] Zhao, J., Sensibaugh, T., Bodenheimer, B., McNamara, T. P., Nazareth, A., Newcombe, N., Minear, M., & Klippel, A. (2020). Desktop versus immersive virtual environments: Effects on spatial learning. *Spatial Cognition & Computation,**20*(4), 328–363. 10.1080/13875868.2020.1817925

