# Supplementary material for: Individual differences in navigation skill: towards reliable and valid measures
Source: Cogn Res Princ Implic. 2025 Jun 7;10:27. doi: 10.1186/s41235-025-00642-5 (PMC12145407; doi:10.1186/s41235-025-00642-5)
Supplement: Supplementary file 1 — Supplementary Material 1. [file 41235_2025_642_MOESM1_ESM.docx]

**Supplement**

**Supplemental Methods**

Two measures were not essential for hypothesis or analyses but were included in the Square Town and Temple Tour paradigms. Before the map-building task for Square Town, participants had to write down a list of objects they remembered from the virtual navigation using the computer. No information about location or order was provided or required in this task. During the Temple Tour paradigm, participants completed the same map-building task a second time immediately following the first map-building activity, this time using an outline of the floorplan to guide their map construction. We performed a two-sample t-test between the blank and the outline map building activity from the Temple Tour paradigm to assess whether the activities would provide distinct measures of map building. However, there was no significant difference between the two activities (p = .59) and we chose not to include the outline map building in analysis.

**Supplemental Results:**


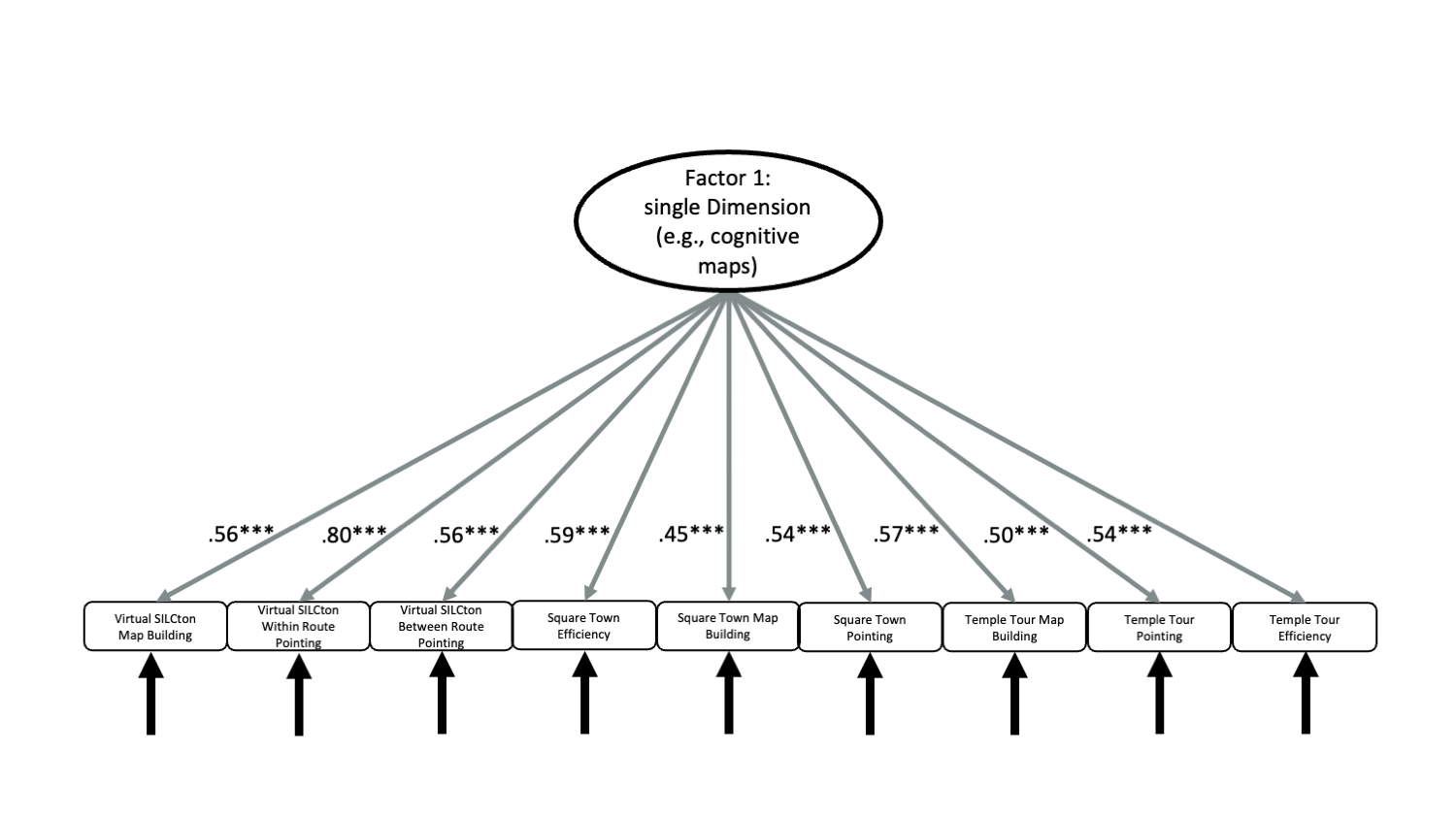


**Supplemental Figure 1:** unifactorial CFA model.

**
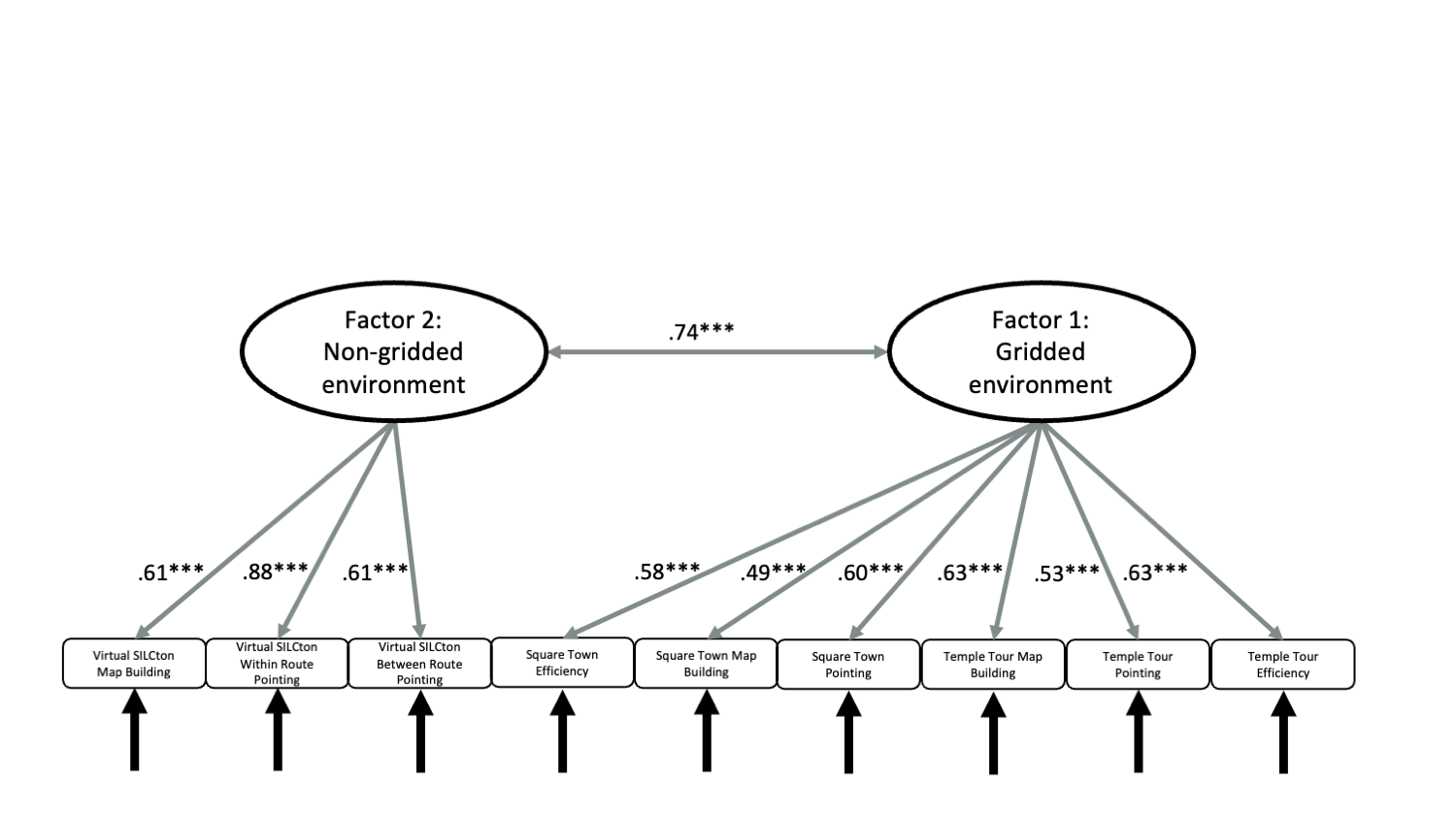
**

**Supplemental Figure 2:** Environment-based bifactorial CFA model.

**
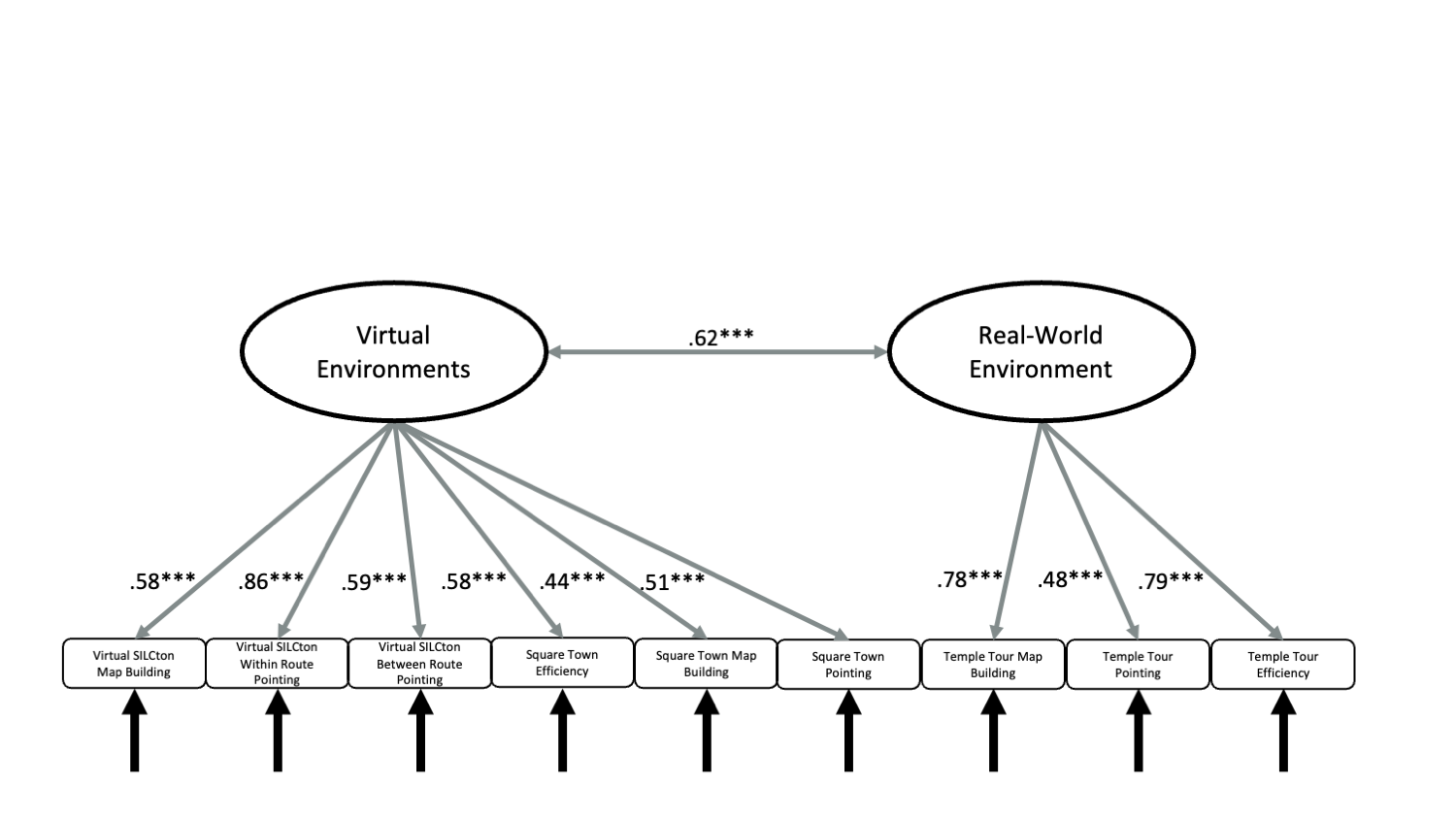
**

**Supplemental Figure 3:** VE vs. RWE bifactorial CFA model.


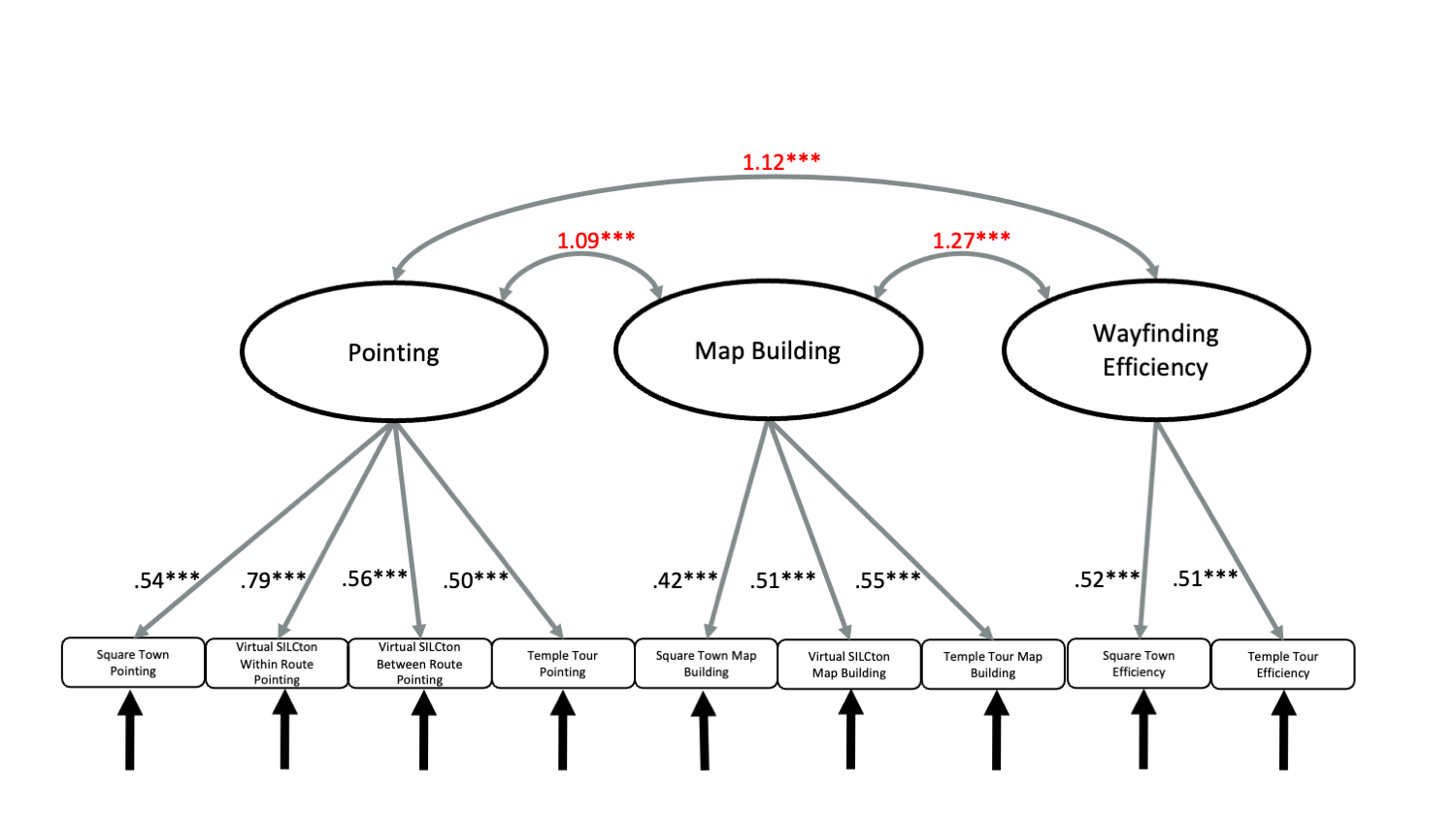


**Supplemental Figure 4:** Task-based CFA: triactorial Model. The model does not make sense for the data given the correlation coefficients greater than 1 shown in red (covariance matrix of latent variables is not positive definite).


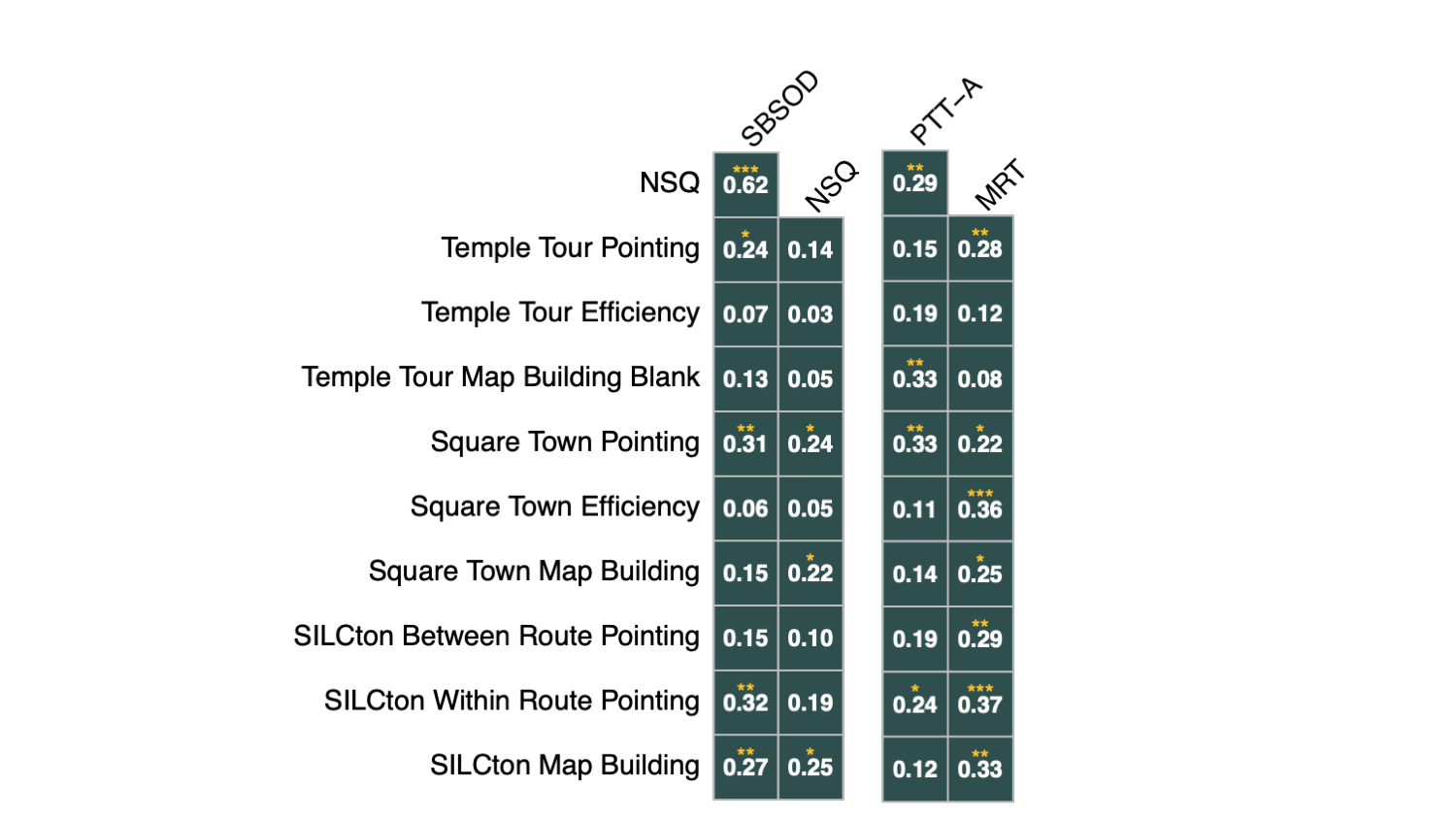


**Supplemental Figure 5:** Partial correlations (controlling for KBIT-IQ and age) between the SBSOD/NSQ and MRT/PTT-A and the navigational measures from each paradigm did not result in any discernible pattern.
